# *N*^4^-Cytosine DNA Methylation Is Involved in the Maintenance of Genomic Stability in *Deinococcus radiodurans*

**DOI:** 10.3389/fmicb.2019.01905

**Published:** 2019-08-21

**Authors:** Shengjie Li, Jianling Cai, Huizhi Lu, Shuyu Mao, Shang Dai, Jing Hu, Liangyan Wang, Xiaoting Hua, Hong Xu, Bing Tian, Ye Zhao, Yuejin Hua

**Affiliations:** ^1^The MOE Key Laboratory of Biosystems Homeostasis & Protection, Zhejiang University, Hangzhou, China; ^2^Department of Infectious Diseases, Sir Run Run Shaw Hospital, College of Medicine, Zhejiang University, Hangzhou, China

**Keywords:** *Deinococcus radiodurans*, genomic stability, DNA methylation, M.DraR1 methyltransferase, differential expression genes

## Abstract

DNA methylation serves as a vital component of restriction-modification (R-M) systems in bacteria, where it plays a crucial role in defense against foreign DNA. Recent studies revealed that DNA methylation has a global impact on gene expression. *Deinococcus radiodurans*, an ideal model organism for studying DNA repair and genomic stability, possesses unparalleled resistance to DNA-damaging agents such as irradiation and strong oxidation. However, details on the methylome of this bacterium remain unclear. Here, we demonstrate that *N*^4^-cytosine is the major methylated form (4mC) in *D. radiodurans*. A novel methylated motif, “C^4m^CGCGG” was identified that was fully attributed to M.DraR1 methyltransferase. M.DraR1 can specifically bind and methylate the second cytosine at *N*^4^ atom of “CCGCGG” motif, preventing its digestion by a cognate restriction endonuclease. Cells deficient in 4mC modification displayed higher spontaneous rifampin mutation frequency and enhanced DNA recombination and transformation efficiency. And genes involved in the maintenance of genomic stability were differentially expressed in conjunction with the loss of M.DraR1. This study provides evidence that *N*^4^-cytosine DNA methylation contributes to genomic stability of *D. radiodurans* and lays the foundation for further research on the mechanisms of epigenetic regulation by R-M systems in bacteria.

## Introduction

In addition to the canonical four-base hereditary information, numerous chemical modifications occur in DNA nucleotides, vastly expanding its structural complexity and information depth (Korlach and Turner, 2012). Among them, DNA methylation is a ubiquitous and pivotal epigenetic phenomenon that occurs in the genomes of all organisms. In bacteria, DNA methylation serves as a vital component of restriction-modification (R-M) systems, which plays a crucial role in defense against foreign DNA (Loenen et al., 2014; Pingoud et al., 2014; Rao et al., 2014; Pleska et al., 2016). It can also modulate the interactions of DNA-binding proteins with their cognate sites and is involved in chromosome replication, correction of DNA mismatches, cell cycle-coupled transcription, and formation of epigenetic lineages (Adhikari and Curtis, 2016; Casadesus, 2016). Recent studies demonstrated that DNA methylation had a global impact on gene expression (Casselli et al., 2018; Kumar et al., 2018).

*N*^6^-methyladenine (6mA), *N*^4^-methylcytosine (4mC), and *C*^5^-methylcytosine (5mC) are the major methylated sites in bacterial genomes, which are generated by methyltransferases (MTases) transferring a methyl group from S-adenosyl-l-methionine (SAM) to certain target bases. To date, the overwhelming majority of sequenced bacterial genomes have been found to encode MTases and are subject to DNA methylation (Murray et al., 2012; Blow et al., 2016). However, the frequency and distribution of methylated bases, as well as the precise sequence targets and biological roles of most MTases remain unknown, largely due to a lack of efficient analytical methods for the detection of these modified bases. Mass spectrometry has long been established as a convenient, rapid and effective technique for qualitative and quantitative analysis of methylated bases (Greer et al., 2015; Schiffers et al., 2017). However, it is unable to achieve genome-wide mapping of target sequences. Fortunately, new cutting-edge methods provide great promise in addressing this issue. Single-molecule, real-time sequencing (SMRT-seq) and nanopore-based sequencing have expanded the epigenetics research toolkit, offering novel methods for the genome-wide, systematic detection of the three forms of methylated DNA and unprecedented opportunities to explore the spectacular landscape of bacterial epigenomes (Clark et al., 2012; Fang et al., 2012; Rand et al., 2017).

*Deinococcus radiodurans* is a robust bacterium with an unparalleled DNA repair system. Experimental evidence has shown that *D. radiodurans* has a similar rate of double-strand break (DSB) formation as *Escherichia coli* under identical conditions (Gerard et al., 2001). However, after receiving a dose of 5,000 gray of γ-irradiation, which introduces approximately 1,600 DSBs per cell, *D. radiodurans* maintains its viability and is able to rebuild its shattered genome within hours (Cox and Battista, 2005). Several mechanisms explain the resistance of *D. radiodurans* to extreme radiation and oxidative stress, including strong self-regulatory systems, cell defense systems and efficient DNA repair mechanisms. In contrast to the long-held assumption that *D. radiodurans* DNA was methyl-deficient due to the unidentified methylated bases or undetectable DNA MTase activity (Ferrandi et al., 2019), the genomic DNA (gDNA) of *D. radiodurans* was reported to contain 6mA and 5mC modifications (Prasad et al., 2005; Patil et al., 2017). The hypomethylation of 6mA leads to dysregulation of metabolic pathways (Shaiwale et al., 2015), whereas 5mC could regulate protein homeostasis in this bacterium (Patil et al., 2017). However, recent experiments using dot-blot and enzyme-linked apta-sorbent assays revealed the absence of 5mC modifications in the gDNA of *D. radiodurans* (Ferrandi et al., 2019).

In the present study, we utilized SMRT-seq combined with sensitive ultra-high-performance liquid chromatography coupled with triple-quadrupole tandem mass spectrometry (UHPLC-QQQ-MS/MS) to analyze DNA methylation patterns in *D. radiodurans*. A novel 4mC modification motif was identified and fully attributable to the presence of the *M.DraR1* gene. M.DraR1 could methylate the target motif and prevent it from being digested by a cognate restriction endonuclease (REase) both *in vivo* and *in vitro*. Cells lacking M.DraR1 were assessed on their varied phenotypes and gene expression patterns. A large number of differentially expressed genes (DEGs) were observed in conjunction with the loss of MTase, including genes involved in DNA repair, transcription and nucleotide processing, suggesting that 4mC methylation is involved in the regulation of gene expression and genomic stability in *D. radiodurans*.

## Results

### *N*^4^-Cytosine Is the Major Methylated Form in *D. radiodurans*

*Deinococcus radiodurans* R1 strain contains five type II and two type IV R-M systems (Supplementary Figure S1 and Supplementary Table S3). A fused R-M enzyme encoded by the open reading frame (ORF) ORF2230P, DraRI was previously characterized as a member of the *Mme*I family recognizing the sequence CAAGN^6*m*^AC and modifying the penultimate adenine (Morgan et al., 2009). ORF14075P encoding MTase belongs to the YeeA (COG1002) methylase, which shares 32% similarity with the protein DrdIV that recognizes and methylates the sequence TACG^6*m*^AC (Morgan et al., 2009). The MTase encoded by ORF15360P contained the typical motifs associated with a 6mA MTase, but its recognition sequence could not be predicted because it lacked the similarity to other MTases with identified specific substrate. ORF16000P encodes a putative MTase, designated as M.DraR1, which has been reported to be an atypical SAM-dependent 5mC MTase for recognizing and methylating random ^5*m*^CpN sites (Patil et al., 2017).

**FIGURE 1:**
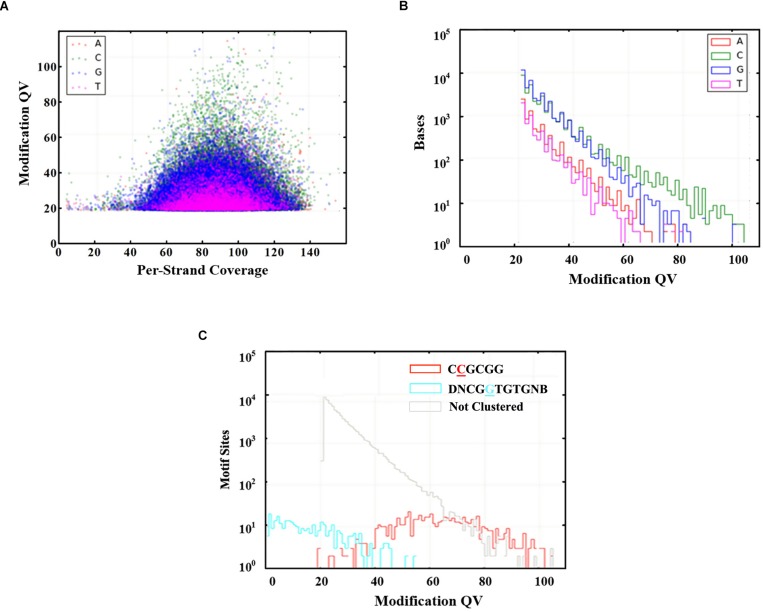
Modified bases in the gDNA of *D. radiodurans* R1. **(A)** Scatter plot of sequencing coverage and kinetic score for all genomic positions. Colors indicate the bases as shown in the upper left of the panel. **(B)** Modification QV Histogram of four type bases modification. **(C)** Modification QV Histogram by motifs. Underlined letters mean the modified positions of each motif.

**FIGURE 2:**
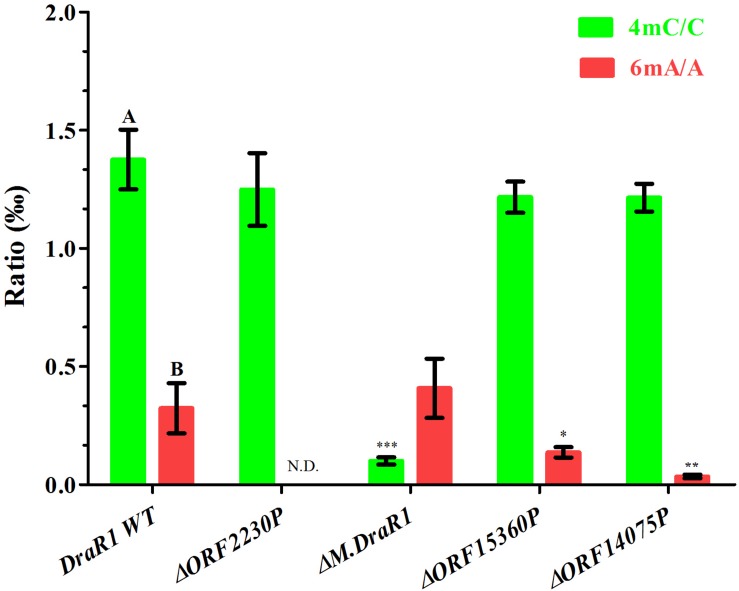
Quantification of 6mA, 4mC, and 5mC in the gDNA of *D. radiodurans* strains using UHPLC-QQQ-MS/MS. The mole ratios are shown. Each column represents the mean and SD of three biological replicates per group. Data with capital letters are significantly different at *p* < 0.01. Compared with DraR1 WT, ^∗^*p* < 0.05, ^∗∗^*p* < 0.01, ^∗∗∗^*p* < 0.001.

Genome-wide analysis of all template positions revealed a cluster of cytosine residues that were clearly separated from the background (Figures 1A,B). Motif analysis identified two MTase motifs, “C^4m^CGCGG” and “DNCGGTGTGNB” with an unknown modification at the fifth base (Figure 1C and Table 1). And no 6mA modification motifs were identified in the gDNA of *D. radiodurans* R1. In addition, the abundance of methylated bases was quantified by UHPLC-QQQ-MS/MS, and results showed that the gDNA of *D. radiodurans* contains approximately 1.3‰ molar ratio of 4mC/C and 0.4‰ of 6mA/A (Figure 2 and Supplementary Figure S2), which corresponds to the results of SMRT-seq analysis. No 5mC modification was detected in this bacterium by either method. Taken together, our study suggests that *N*^4^-cytosine is the major methylated residue in *D. radiodurans*.

**TABLE 1:** Comparison of genome-wide methylation patterns of *D. radiodurans* with *M.DraR1* mutant strain.

**Strain**	**Motifs**	**Modified position**	**Type**	**% Motifs detected**	**# Of Motifs detected**	**# Of motifs in genome**	**Mean modification QV**	**Mean motif coverage**	**Partner motif**
DraR1^a^	CCGCGG	2	m4C	95.83%	667	696	64.24	80.95	CCGCGG
	DNCGGTGTGNB	5	Unknown	16.57%	59	356	39.15	82.69	None
Δ*M.DraR1*^b^	GNNNNNH	1	Unknown	17.78%	265908	1495418	39.04	106.59	None
	GSVVNVNG	1	Unknown	11.98%	35310	294745	37.22	108.43	None
	GAGVNGBGV	1	Unknown	6.65%	516	7761	37.13	106.09	None
	CWNNNVNH	1	m4C	3.14%	14225	453199	43	109.41	None
	TCR	1	Unknown	1.82	4327	237866	33.95	118.77	None

*^*a*^DraR1, *Deinococcus radiodurans* R1 wild type strain. NCBI accession numbers: CP015081 to CP015084. ^*b*^Δ*M.DraR1, D. radiodurans* R1 deletion *M.DraR1* strain. NCBI accession numbers: CP031500 to CP031503.*

### M.DraR1 Is a Novel α-Class *N*^4^-Cytosine Methyltransferase

M.DraR1, located at the small plasmid of genome (A2G07_16000 or DR_C0020) in *D. radiodurans*, is predicted to be a typical α-class DNA MTases containing a canonical N-terminal SAM-binding motif (“FxGxG”) followed by a centrally located target recognition domain and a C-terminal catalytic motif (“SPPY”). Residues in M.DraR1, including V44, D63, P64, T71, G79, K98, T255, N261, G394, and R404, are also moderately conserved in its homologs (Supplementary Figure S3), indicating that these residues might play important roles in MTase activities. Sequence analysis revealed that M.DraR1 is an *N*^6^-adenine/*N*^4^-cytosine methylase. However, the target sequence context, the type and exact site of methylation are still unclear.

To rigorously assess the ability of M.DraR1 transferring a methyl group to either *N*^4^-cytosine or *N*^6^-adenine, we generated an *M.DraR1* gene knockout strain (Supplementary Figures S4A,B). The other three DNA MTase genes were also deleted (Supplementary Figures S5A–C). Compared to wild type *D. radiodurans*, deletion of the *M.DraR1* gene drastically decreased the molar ratios of 4mC/C, but had no effect on the abundance of 6mA (Figure 2), suggesting that M.DraR1 is an *N*^4^-cytosine DNA MTase but not the *N*^6^-adenine MTase. Kinetic signatures obtained from SMRT-seq showed the complete loss of the second 4mC methylation site in the sequence “CCGCGG” of Δ*M.DraR1* mutant (Table 1). The other three DNA MTases were confirmed to be involved in 6mA modification. These results suggest that M.DraR1 specifically recognizes the target sequence “CCGCGG” and methylates the *N*^4^ atom of the second cytosine in *D. radiodurans*, indicating it is a novel α-class *N*^4^-cytosine MTase.

### M.DraR1 Specifically Methylates and Protects the “CCGCGG” Motif From Cleavage by a Cognate REase

**FIGURE 3:**
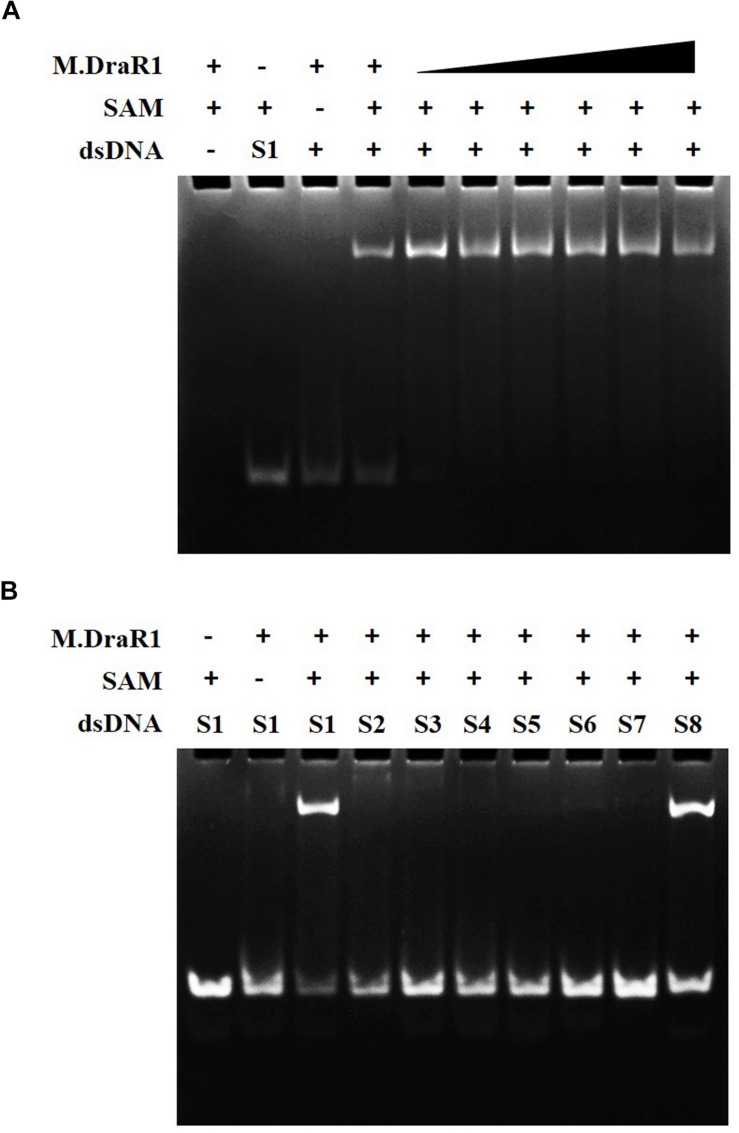
EMSA analysis of DNA binding by M.DraR1. **(A)** M.DraR1 binding oligonucleotide cassettes (annealed by S1-F and S1-R oligonucleotides in Supplementary Table S2) containing a “CCGCGG” sequence with different concentrations (from 100 μM to 1 mM). Excess (0.5 mM) or none SAM were added in binding buffer (20 mM Tris–HCl pH 8.0, 100 mM KCl, 7 mM β-Me, 0.1mM EDTA and 0.1 mg/mL BSA). **(B)** M.DraR1 could not bind oligonucleotides with site mutations in the “CCGCGG” sequence (Supplementary Table S2, from Supplementary Tables S2-S7 paired oligomers). Another blunt-ended dsDNA for “CCGCGG” was analyzed as well (last lane, S8 oligonucleotide cassette). The 100 μM double strand cassettes were used for each reaction. All the reactions were performed in three independent biological replicates.

M.DraR1 protein was purified by stepwise affinity column purification as described in “Materials and Methods” section (Supplementary Figures S6A–C). And the purified recombinant protein was confirmed to be the product of ORF16000 (Supplementary Figures S6D,E and Supplementary Table S4). M.DraR1 exists mainly in its monomeric state in solution during the size exclusion column (Supplementary Figures S6F,G), which is consistent with that of previously reported DNA MTases (Ma et al., 2016). To test the DNA binding capacity of the M.DraR1 protein, different oligonucleotides were generated by annealing corresponding single strands (Supplementary Table S2). As shown in Figure 3, M.DraR1 was able to bind oligonucleotides containing “CCGCGG” in the presence of SAM (Figure 3A), and the binding was saturated by increasing the molar ratio of M.DraR1 to oligonucleotides (Figure 3A). Furthermore, M.DraR1 was unable to bind oligonucleotides containing any mutations (Figure 3B), which indicates the high specificity of M.DraR1.

**FIGURE 4:**
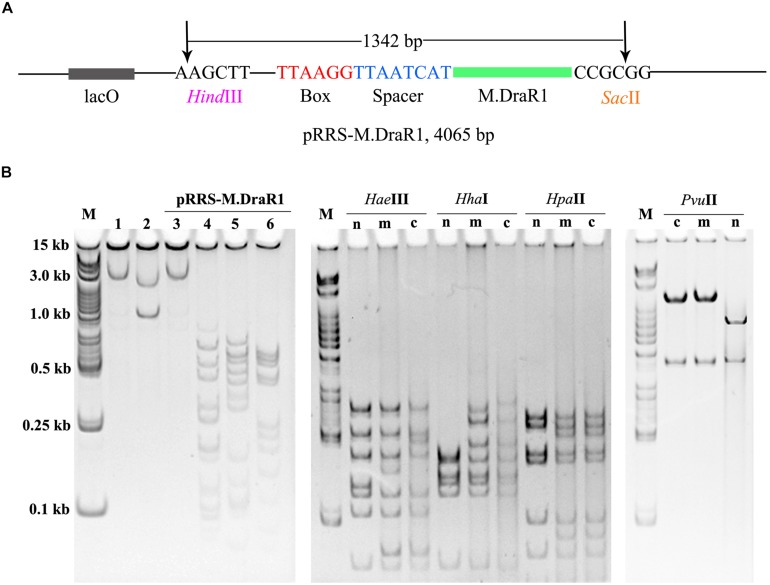
Confirmation of activity of M.DraR1 using methylation protection assays. **(A)** pRRS – M.DraR1, a high-copy replicon that contains CCGCGG motif and expresses M.DraR1 was constructed. Black arrow stands for the restriction sites of *Hin*dIII and *Sac*II, respectively. **(B)** Analysis of M.DraR1 methylation *in vivo*. pRRS-M.DraR1 and pRRS null vectors (n) were transformed into methylation-deficient *E. coli* ER2796 cells, and plasmid DNA were recovered from stationary phase cultures after growth at 37°C for about 20 h. Un-methylated control substrate of pRRS-M.DraR1 was generated by PCR amplification. *Hin*dIII predigested pRRS-M.DraR1 from ER2796 (m) (lane 1) prevented digestion by *Sac*II (lane 3), but not by *Hae*III (lane 4), *Hha*I (lane 5), and *Hpa*II (lane 6) in contrast to PCR amplified pRRS-M.DraR1 (c) that is digested by *Sac*II (lane 2). All these substrates showed similar digestive profiles by *Hae*III, *Hha*I, *Hpa*II, and *Pvu*II. M, 250 bp DNA Ladder (TSJ105-100) from Beijing TsingKe Biotech Co., Ltd. All experiments were performed in three independent biological replicates.

**FIGURE 5:**
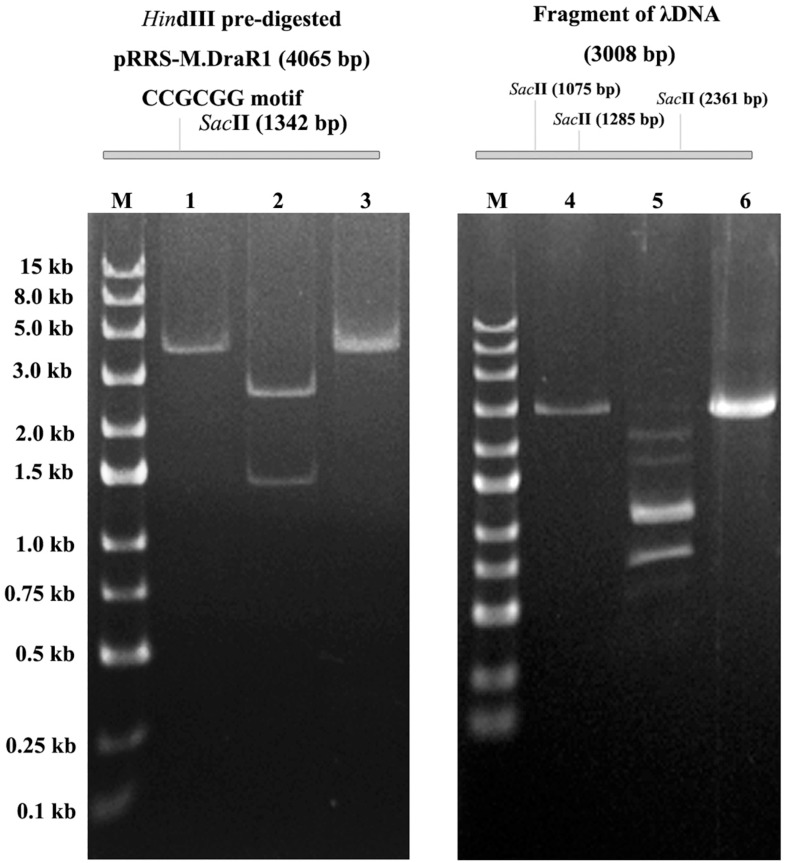
*In vitro* methyltransferase activity assay of the purified M.DraR1. PCR amplified pRRS-M.DraR1 vector was linearized by *Hin*dIII (lane 1), and then purified and methylated with M.DraR1 enzyme *in vitro*. Methylation of the plasmid prevented it digestion by *Sac*II (lane 3) in contrast to un-methylated control one (lane 2). The methylation of PCR amplified λDNA fragment containing three “CCGCGG” sites (lane 4) with M.DraR1 protected it from being digested (lane 6) by *Sac*II as well. Lane 5 is the unmethylated λDNA fragment. M, 250 bp DNA ladder (TSJ105-100) from Beijing TsingKe Biotech Co., Ltd. All experiments were performed in three independent biological replicates.

To further explore the specificity of M.DraR1 and assess its ability to effectively inhibit REases, restriction analyses were carried out using the pRRS-M.DraR1 vector (Figure 4A). Methylated pRRS-M.DraR1 plasmid could be digested by *Hin*dIII (“AA^↓^GCTT”) but not by *Sac*II (a relevant cognate REase), both of them are sensitive to cytosine modifications (Figure 4B). These results indicate that M.DraR1 cannot methylate “AAGCTT” sites at C residues *in vivo* but does specifically methylate “CCGCGG” motifs. Other four restriction enzymes with defined specific sequences (CAG^↓^CTG for *Pvu*II, GCG^↓^C for *Hha*I, C^↓^CGG for *Hpa*II, and GG^↓^CC for *Hae*III), which are resistant to cleavage by cytosine methylation at CpN sites, were also analyzed. pRRS-M.DraR1 and its PCR products exhibited similar gel patterns to that of pRRS null vector (Figure 4B), which suggests that M.DraR1 cannot randomly methylate CpN sites *in vivo*. MTase activity of M.DraR1 *in vitro* showed that those DNA substrates were resistant to cleavage by *Sac*II but completely digested by other enzymes (Figure 5 and Supplementary Figure S7). These results also suggested that recombinant M.DraR1 could specifically methylate “CCGCGG” motifs *in vitro* and protect them from cleavage.

### Cells Devoid of M.DraR1 Causes Genomic Instability

**FIGURE 6:**
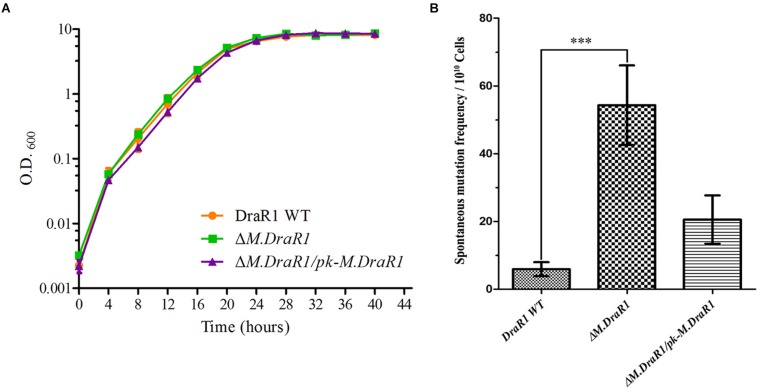
Deletion M.DraR1 gene does not affect the growth rate but induces higher spontaneous rifampin mutant frequency in *D. radiodurans* R1. **(A)** Growth curves of *D*. *radiodurans* strains. Wild-type, Δ*M.DraR1* mutant and the *M.DraR1* complementary strains were individually grown in TGY medium, and growth rates were recorded by measuring the OD_600_ every 4 h. **(B)** The spontaneous rifampin mutant frequency of *D. radiodurans* strains. The spontaneous mutant frequency is the number of rifampin resistant colonies divided by the total number of viable cells. Averages and standard deviations were calculated from three independent experiments. Compared with DraR1, ^∗∗∗^*p* < 0.001.

Deletion of the *M.DraR1* gene did not significantly affect cell growth under optimal conditions (Figure 6A). The *D. radiodurans M.DraR1* mutant showed increased spontaneous rifampin mutation frequency (Figure 6B). Compared to the wild type, the mutant showed a ∼ninefold increase significantly (*p* = 0.002), suggesting that M.DraR1 mediated DNA 4mC methylation might be involved in the genomic stability in *D. radiodurans*. Furthermore, cells lacking *M.DraR1* led to a >100-fold increase in transformation frequency (Table 2). And the relative intermolecular recombination efficiency of the mutant strain was approximately ∼39-fold higher than that of the wild type strain (Table 2). Compared with the pRADK vector, the modified plasmid pRADKm caused reduced transformability, while the use of methylated M.pRADKm plasmid with M.DraR1 lead to an efficient improvement of DNA transformation frequencies in *D. radiodurrans* strains (Table 3). These results suggest that the spontaneous rifampin mutation frequency, recombination capacity and transformability of *D. radiodurans* might be regulated by 4mC modification. Thus, we proposed that 4mC methylation could act as a methylation barrier against foreign DNA, contributing to genomic stability.

**TABLE 2:** Transformation and recombination frequency of *D. radiodurans* and *M.DraR1* mutant strains.

**Strain**	**Transformation efficiency (10^14)a^**	**Intermolecular recombination frequency (10^11^)^b^**	**Relative intermolecular recombination efficiency (10^2^)^c^**
			
DraR1	7.07 ± 1.13	1.78 ± 0.23	2.52 ± 0.32
Δ*M.DraR1*	(4.39 ± 0.57) × 10^3**^	(4.30 ± 1.70) × 10^4*^	97.85 ± 38.69^∗^

*^*a*^The transformation efficiency is the number of transformant colonies divided by the total number of viable cells. 0.2 μg pRADK plasmid were transformed into each strain. Mean and SD were calculated from three independent experiments. ^*b*^0.5 μg of Δ*crtB:Kana*^+^ fragment DNA were transformed and the intermolecular recombination frequency is the number of recombinated colonies (white colones were calculated on TGY plates containing 20 mg/mL kanamycin) divided by the total number of viable cells. ^*c*^To allow for transformation efficiency, the relative intermolecular recombination efficiency of each strain is calculated by intermolecular recombination frequency divided by the transformation efficiency. Compared with DraR1, ^∗^*p* < 0.05, ^∗∗^*p* < 0.01.*

**TABLE 3:** Methylation by M.DraR1 is required for DNA transformation of *D. radiodurans* strains.

**Groups**	**Number of transformant colonies (×10^3^ CFU/mL)**	**Total number of viable cells (×10^16^ CFU/mL)**	**Transformation efficiency (×10^14^)**
			
DraR1 + pRADKm^1^	3.65 ± 0.92	17.67 ± 5.51	2.07 ± 0.37 ^A^
DraR1 + M. pRADKm^2^	23.17 ± 3.69	19.60 ± 3.39	11.82 ± 1.88 ^B^
Δ*M.DraR1* + pRADKm	(2.90 ± 0.10) × 10^3^	35.47 ± 5.70	(8.18 ± 0.28) × 10^2**a^
Δ*M.DraR1* + M. pRADKm	(3.77 ± 0.68) × 10^3^	1.00 ± 0.28	(3.77 ± 0.88) × 10^4*b^

*^1^pRADKm, a plasmid derived from pRADK by site-directed mutagenesis, contains one “CCGCGG” site. ^2^M.pRADKm, the methylated pRADKm plasmid by M.DraR1 enzyme *in vitro*. Compared with pRADKm transformation, data with capital letters are significantly different at *p* < 0.01, lowercase at *p* < 0.05. Compared with DraR1 group, ^∗^*p* < 0.05, ^∗∗^*p* < 0.01.*

### Absence of *N*^4^-Cytosine DNA Methylation Leads to Expression Changes of Proteins Involved in DNA Damage Response

**FIGURE 7:**
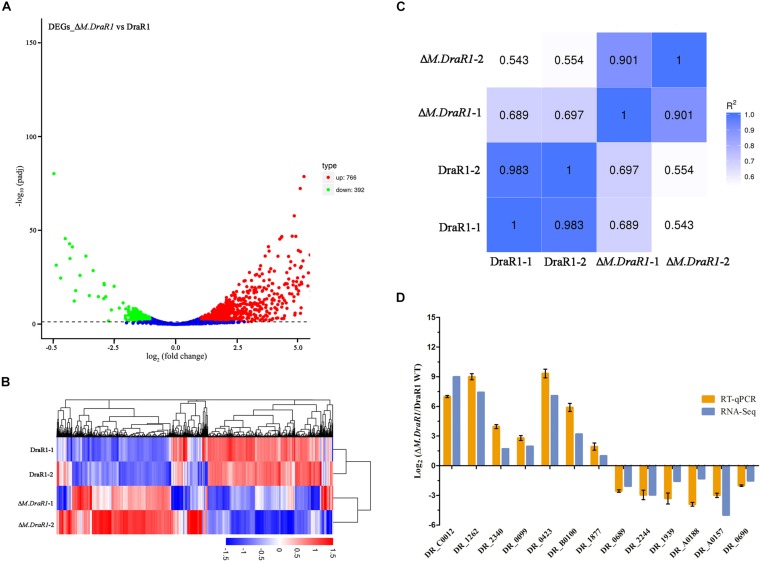
Comparison of differentially expressed genes (DEGs) identified between *D. radiodurans* R1 and Δ*M.DraR1* mutant strains. **(A)** Volcano Plot analysis of DEGs in Δ*M.DraR1* strain compared with *D. radiodurans* wild type. Genes with an adjusted *p* < 0.05 found by DEGs analytical method were assigned as differentially expressed. **(B)** Hierarchical clustering of DEGs in each sample. Blue bands indicate a low gene expression level, and red bands indicate a high gene expression level. **(C)** Pearson correlation between these strains. The correlation coefficient of FPKM was >90.1% between two independent biological replicates of each sample. **(D)** RT-qPCR validation of the expression levels of 13 candidate genes. Error bars indicate the standard deviation of averages from three biological replicates.

RNA-seq was used for differential expression gene analysis between wild-type and Δ*M.DraR1* strains. A total of 1,158 genes (766 upregulated genes and 392 downregulated genes) were differentially expressed in Δ*M.DraR1* strain (Figure 7A). Hierarchical clustering was performed to determine the expression patterns for all DEGs and samples were clustered into two groups (Figure 7B). The correlation coefficient of FPKM was >90.1% between two independent biological replicates of each group (Figure 7C), indicating that the replicates were highly correlated and credible. These results were also validated by RT-qPCR (Figure 7D).

**FIGURE 8:**
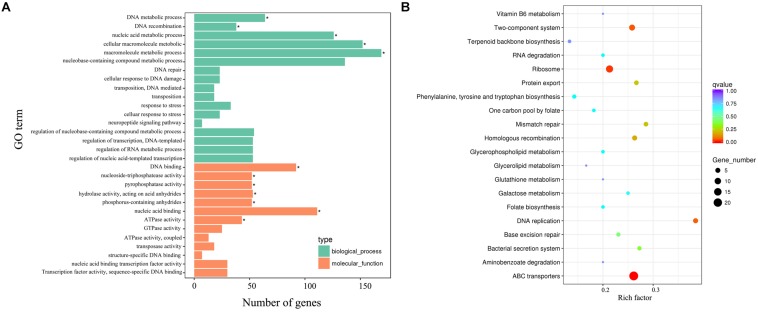
The biological relationship of the upregulated DEGs. **(A)** Gene ontology (GO) annotations of the upregulated unigenes. The GO term (left *y*-axis) and the number of DEGs (down *x*-axis) are shown. Bars with asterisks represent significantly enriched terms (*p* adjust < 0.05). **(B)** KEGG functional annotations of these upregulated unigenes. The scatter diagram shows the enrichment of DEGs in the signaling pathways.

Strikingly, gene ontology (GO) enrichment analysis revealed that upregulated unigenes were significantly enriched in twelve subcategories (Figure 8A). The majority of unigenes in the biological process category were associated with macromolecule metabolic processes. Interestingly, a number of unigenes were also associated with DNA recombination. Within the molecular function category, most unigenes were assigned to nucleic acid, DNA binding and hydrolase, and nucleoside triphosphatase activity. The KEGG enrichment analytical scatter diagram showed that the major pathways affected in the deletion strain belonged to ABC transport, two-component systems, DNA replication, homologous recombination, and protein export pathways (Figure 8B). Two-component systems and homologous recombination are the most reported pathways involved in the robust DNA repair capacity of *D. radiodurans*. In contrast, GO and KEGG pathway enrichment analysis showed that downregulated unigenes were mainly involved in energy or small-molecule metabolic processes, such as the tricarboxylic acid cycle, glycolysis, microbial metabolism in diverse environments, and sulfur metabolism (Supplementary Figure S8).

Interestingly, the majority of DNA damage response genes, such as *ddrA*, *ddrB*, *ddrO*, *ddrG*, *ddrJ*, *ddrK*, *ddrD*, and *pprA*, were significantly upregulated (Table 4). Several genes involved in DNA recombinational repair pathway including *recA*, *recO*, *rexD*, and *ruvB* were also significantly upregulated. Putative competence genes, including those encoding prepilin peptidase, CRP and CinA, were upregulated in the mutant strain. This gene expression pattern of the Δ*M.DraR1* strain resembled that of *D. radiodurans* during recovery after exposure to acute radiation (Liu et al., 2003), indicating that the absence of *N*^4^-cytosine DNA methylation may result in intracellular stress.

**TABLE 4:** Expression changes of proteins involved in DNA damage response in *N*^4^-cytosine methylation deficient strain.

**Gene ID**	**Log_2_ fold**	***p*-value**	**Function annotation**
**Ddr and Ppr proteins**			
DR_0423	7.088	2.53E-125	Single-stranded DNA-binding protein, DdrA
DR_0070	4.8588	1.56E-58	Single-stranded DNA-binding protein, DdrB
DR_0003	4.993	1.80E-25	Hypothetical protein, DdrC
DR_0326	5.9294	7.20E-80	Hypothetical protein, DdrD
DR_0227	6.7254	2.40E-68	Hypothetical protein, DdrG
DR_0438	2.8941	1.45E-15	Hypothetical protein, DdrH
DR_1263	6.2741	5.17E-81	Hypothetical protein, DdrJ
DR_1264	5.7875	1.67E-55	Hypothetical protein, DdrK
DR_2574	3.6669	4.24E-37	Transcriptional repressor of the RDR regulon, DdrO
DR_B0100	3.2083	1.08E-18	Putative RNA or DNA ligase, DdrP
DR_A0346	3.4373	2.42E-26	DNA damage repair protein, PprA
**DNA repair proteins**			
DR_1262	7.4276	1.96E-155	Ribonucleoprotein Ro/SS-A-like protein
DR_B0099	3.3137	5.88E-18	Poly ADP-ribose glycohydrolase, PARG
DR_1297	3.09	6.24E-12	Uncharacterized membrane protein, MutK
DR_0221	2.7957	1.35E-07	Endonuclease domain-containing protein (DUF559)
DR_0596	2.6105	2.51E-14	DNA helicase subunit, RuvB
DR_1902	2.6449	2.96E-11	ATP-dependent RecD-like DNA helicase, RecD2
DR_2586	2.4528	0.0001281	Exonuclease VII small subunit, XseB
DR_1039	2.3424	1.31E-11	DNA mismatch repair protein, MutS
DR_0899	2.0659	3.07E-06	Ribonuclease HI, RnhA
DR_1310	1.8549	6.43E-05	SOS response regulatory protein, OraA/RecX
DR_B0098	1.9345	3.71E-08	Polynucleotide kinase, PNKP
DR_0428	1.6244	0.00097529	Alkylated DNA nucleotide flippase, Atl1
DR_0491	2.0405	5.13E-07	Methylglyoxal and glyoxal deglycase
DR_0099	1.9713	3.45E-08	Single-stranded DNA-binding protein, SSB
DR_1696	2.0243	6.48E-10	DNA mismatch repair ATPase, MutL
DR_2340	1.7145	1.07E-09	Recombinase A, RecA
DR_1913	1.6998	6.16E-08	DNA gyrase subunit A, GyrA
DR_0928	1.5242	5.73E-05	Endonuclease III, Nth
DR_2584	1.3687	0.0027148	AlkA
DR_0819	1.2406	0.0031834	DNA repair protein, RecO
DR_1572	1.0071	0.00084984	DNA helicase IV, HelD
DR_1374	–1.0828	0.00017952	DNA topoisomerase IA
DR_B0136	–1.1809	0.0065165	Superfamily II DNA or RNA helicase, HepA
DR_2444	–1.1741	0.00023378	HRDC-domain containing protein
DR_2438	–1.1513	0.0042907	Endonuclease III, Nth
DR_A0188	–1.3045	1.07E-05	ATPase subunit, UvrA2
**Nucleoid-associated proteins**			
DR_2263	–1.7121	1.15E-05	starvation-inducible DNA-binding protein, Dps-1
DR_A0065	–1.1282	0.00034972	DNA-binding protein HU-beta, Hu
DR_0689	–2.0547	0.00070292	Uracil DNA glycosylase, Ung
DR_0690	–1.5222	0.000211	DNA topoisomerase IB, Top1
DR_1913	1.6998	6.16E-08	DNA gyrase subunit A, GyrA
DR_0906	1.012	0.00039493	DNA gyrase subunit B, GyrB
**Putative competence genes**			
DR_2065	1.37	0.00038418	Prepilin peptidase, type IV
DR_0997	2.1888	6.36E-06	CRP family transcriptional regulator
DR_1646	2.1841	8.93E-08	cyclic AMP receptor protein
DR_2338	2.5946	6.05E-22	CinA protein
DR_B0067	–2.9352	1.8467E-22	Extracellular nuclease
**Stress resistance-associated regulatory proteins**			
DR_C0012	8.9928	1.35E-43	DNA-binding response regulator
DR_2574	3.6669	4.24E-37	Transcriptional repressor of the RDR regulon
DR_A0074	2.6564	2.30E-11	LexA-XRE SOS regulon
DR_2416	2.4366	1.79E-16	DrtS DNA damage response TCS sensor
DR_0171	2.2432	3.55E-09	Putative transcriptional regulator
DR_0892	1.9984	2.07E-08	Sensor histidine kinase
DR_2415	1.6885	4.09E-06	DNA damage response TCS regulator, DrtR
DR_A0344	1.5513	0.0006724	LexA-ArsR SOS regulon
DR_0987	1.2235	0.00073958	Deinococcus QS regulator, DqsR
DR_1174	–1.0052	0.00072146	Extracellular sensor domain CHASE1
DR_2244	–2.9471	1.99E-14	Phosphate regulon sensor protein, PhoR

## Discussion

The complete genome of *D. radiodurans* contains two circular chromosomes and two circular plasmids with a GC content >66.3% (Hua and Hua, 2016). Despite that the gDNA of *D. radiodurans* was previously assumed to be methyl-deficient (Ferrandi et al., 2019), Patil et al. detected 6mA and 5mC bases in its gDNA (Prasad et al., 2005; Patil et al., 2017). However, our SMRT-seq and UHPLC-QQQ-MS/MS results suggest that 4mC is the dominant methylated base in *D. radiodurans*, while the abundance of 6mA is significantly lower and 5mC is undetectable. These results are consistent with those of Ferrandi et al. (2019), who failed to identify any 5mC methylated bases in gDNA of this bacterium. Thus, the *N*^4^-cytosine DNA methylation appears to be the epigenetic marker in *D. radiodurans*.

The comparative analysis suggested that MTases encoded by ORF2230P, ORF14075P and ORF15360P are involved in 6mA modification in *D. radiodurans*, whereas only the MTase encoded by ORF16000P was implicated in 4mC methylation. And the protein encoded by ORF10260P (*DR_0643*) was annotated as an RsmD-like RNA methyltransferase, which was once proposed to be a 6mA DNA MTase (Shaiwale et al., 2015). As a member of the *Mme*I family, DraRI in *Deinococcus* was reported to possess no DNA methyltransferase activity *in vivo*, but displayed endonuclease activity once disrupted ORF was corrected (Morgan et al., 2009). Levels of 6mA in our study were undetectable by UHPLC-QQQ-MS/MS after deletion of this gene, indicating that DraRI has MTase activity.

The S/DPPY/F motifs are the hallmark of *N*^4^-cytosine MTase active sites (Malone et al., 1995; Bujnicki and Radlinska, 1999). In contrast, an invariant Pro-Cys dipeptide (PC motif) is present in the active site of 5mC DNA MTases (Kumar et al., 1994). M.DraR1 contains SPPY and FxGxG motifs and but no other conserved motifs such as PC, ENV, QRR, RE or GN, suggesting that M.DraR1 is not likely a 5mC MTase. In the current study, deletion of *M.DraR1* drastically reduced 4mC content and resulted in complete loss of the modified “C^4m^CGCGG” motif, suggesting that M.DraR1 is an *N*^4^-cytosine MTase. Electromobility shift assay (EMSA) also confirmed that M.DraR1 can rigorously bind to DNA substrates containing the sequence “CCGCGG” in the presence of SAM. Restriction digestion pattern analysis *in vivo* and *in vitro* provided further evidence that M.DraR1 is able to protect DNA substrates from *Sac*II digestion. Taken together, these results indicated that M.DraR1 is an α-class *N*^4^-cytosine MTase, which methylates the *N*^4^ atom rather than *C*^5^ position of the second cytosine in “CCGCGG” motifs (Roberts et al., 2015).

Despite the low frequency compared to that of 6mA, 4mC is a prevalent modified base in thermophilic bacterial DNA and also occurs in many mesophilic bacteria (Ehrlich et al., 1985). A recent study revealed that *N*^4^-cytosine DNA methylation in *Helicobacter pylori* was involved in transcriptional regulation and pathogenesis, suggesting that 4mC is not only a component of R-M systems but also serves as a global epigenetic regulator (Morgan et al., 2016; Kumar et al., 2018). In our study, 4mC plays a crucial role in the maintenance of genomic stability in *D. radiodurans*, as higher spontaneous rifampin mutation frequency, enhanced recombination and transformation efficiencies were observed in *N*^4^-cytosine methylation deficient strains.

Our observations raise the question of why 4mC modifications are the epigenetic marker in *D. radiodurans*. 4mC has been observed more often in thermophilic bacteria than in non-thermophilic bacteria (Ehrlich et al., 1985; Beaulaurier et al., 2019). This may be related to the fact that *N*^4^-methylated cytosine is more resistant to heat-induced deamination, which can introduce disadvantageous T:G mismatches into the genome compared with *C*^5^-methylated cytosine (Ehrlich et al., 1985). Therefore, we speculate that *D. radiodurans* inherited 4mC methylation from the shared ancestor of *Deinococcus-Thermus*. We also noted that some other *Deinococcus* species also possess 4mC and have few 5mC residues in their gDNA (data are not shown), which is consistent with the findings of Blow et al. (2016) who also identified 4mC but no 5mC modifications in three other *Deinococcus-Thermus* species. Another interesting possibility is that this bacterium may have acquired the MTase gene via horizontal gene transfer since M.DraR1 shows the highest sequence homology with M.Rca13941ORF67P (43% identity and 58% positivity), a type II *N*^4^-cytosine DNA MTase, identified from *Roseiflexus castenholzii* DSM13941, were found to recognize the “CCGCGG” motif as well (Roberts et al., 2015).

Natural transformation, the stable uptake and heritable integration of extracellular DNA, plays a major role in genomic diversity, ecological diversification and functional regulation (Thomas and Nielsen, 2005; Johnsborg et al., 2007). In *D. radiodurans*, the absence of m4C affected the natural transformation pathway through epigenetic regulation of gene expression. This is consistent with our findings that several homologous competence genes, including the genes encoding prepilin peptidase, CRP and CinA were upregulated and the gene encoding extracellular nuclease downregulated in the Δ*M.DraR1* strain. Furthermore, uptake of pRADKm DNA was reduced, whereas M.pRADKm was enhanced in both *D. radiodurans* wild type and Δ*M.DraR1* mutant strains, suggesting that there might be a relevant cognate REase limiting the foreign DNA (containing “CCGCGG” motifs) uptake in *D. radiodurans*. A putative GIY-YIG endonuclease encoded by ORF15945P was also annotated in the genome of *D. radiodurans*, which may recognize and cleave the same motif based on the high similarity of its amino acid sequence with that of R.Eco29kI.

Cells lacking 4mC methylation displayed decreased genomic stability in *D. radiodurans*. Previous studies proposed that DNA methylation might affect the chromosome topology whereby induces conformational changes to a bacterial genome, rendering previously inaccessible genes accessible to the transcriptional machinery (Polaczek et al., 1998; Ngo et al., 2016; Beaulaurier et al., 2019). Interestingly, almost all nucleoid-associated proteins, e.g., Dps-1, HU, UDG, and type IB topoisomerase, were found to be significantly downregulated in the Δ*M.DraR1* strain, suggesting the presence of defects in DNA compaction in the mutant strain (Blasius et al., 2008; Nguyen et al., 2009; Reon et al., 2012; Wang et al., 2012). DNA gyrase, containing GyrA and GyrB, which are involved in DNA relaxation, decatenation and supercoiling (Kota et al., 2016), was upregulated in Δ*M.DraR1*, implying the role of 4mC methylation in the regulation of DNA condensation. The “CCGCGG” sites also found to be uniformly distributed across the *D. radiodurans* genome, indicating that they may also affect DNA compaction through epigenetic modulation of gene expression in *D. radiodurans*.

## Conclusion

We characterized *N*^4^-cytosine DNA methylation in the extremophile *D. radiodurans* R1 and addressed its possible association with transcriptional expression patterns. Evidence from our study suggested that M.DraR1 is a novel α-class *N*^4^-cytosine MTase that specifically recognizes the target sequence “CCGCGG” and methylates the second cytosine at the *N*^4^ position, preventing it from being digested by cognate endonucleases both *in vitro* and *in vivo*. Moreover, cells lacking 4mC DNA modifications exhibited higher spontaneous mutation frequencies and enhanced recombination and transformation efficiencies. And a large number of DEGs were observed in conjunction with the loss of the MTase enzyme. Our study has provided new insights into the properties and roles of *N*^4^-cytosine DNA methylation in the maintenance of genomic integrity in *D. radiodurans*.

## Materials and Methods

### Strains and Growth Conditions

All strains and plasmids used in this study are listed in Supplementary Table S1. *E. coli* strains were grown in Luria-Bertani (LB) liquid media or on agar (1.5% Bacto-agar) plates supplemented with appropriate antibiotics at 37°C with aeration. All *D. radiodurans* strains were grown in tryptone glucose yeast extract (TGY) liquid media or on agar plates (0.5% tryptone, 0.1% glucose, and 0.3% yeast extract) supplemented with appropriate antibiotics at 30°C with aeration.

### SMRT-Seq

Genomic DNA was extracted using a QIAamp DNA mini kit (Qiagen, Valencia, CA, United States) following the manufacturer’s protocol. The DNA quality was determined using gel electrophoresis and a NanoDrop 2000 spectrophotometer (NanoDrop Technologies, Wilmington, DE, United States). SMRT sequencing and *de novo* assembly were carried out as described previously (Hua and Hua, 2016). DNA modification detection and motif analysis were performed using the standard settings in the “RS Modification and Motif Analysis.1” protocol included in SMRT Portal version 2.2.0.^1^ Modified sites were then identified through kinetic analysis of the aligned DNA sequence data and grouped into motifs using Motif Finder (v1)2 (Clark et al., 2012; Blow et al., 2016). The SEQWARE computer resource was used to annotate R-M system genes (Blow et al., 2016). The results were stored into REBASE (Roberts et al., 2015). PacBio motif files and sequencing data were submitted to the NCBI database (Table 1).

### M.DraR1 Mutant Strain Construction and Complementation

Tripartite ligation and double-crossover recombination methods were used for gene mutation as described previously with slight modifications (Dai et al., 2018). Briefly, upstream and downstream fragments of the target gene were PCR-amplified, digested and ligated to a streptomycin resistance cassette. The tripartite ligated fragment were PCR-amplified using P1/P4 primers and transformed into *D. radiodurans* competent cells. Mutant colonies were screened on TGY plates containing 10 μg/mL streptomycin (TS plate) and confirmed by PCR analysis and DNA Sequencing. The mutant strain was named Δ*M.DraR1*.

For gene complementation, the full-length *M.DraR1* gene fragment was PCR-amplified using M.DraR1-F and M.DraR1-R primers and ligated into the shuttle vector pRADK to construct the complementation plasmid pRAD-M.DraR1. After confirmation by sequencing using primers pRAD-F and pRAD-R, the plasmid was subsequently transformed into Δ*M.DraR1* complement cells and selected on TGY plates containing streptomycin and 4 μg/mL chloramphenicol (TSC plate). The resulting strain was designated Δ*M.DraR1*/*pk-M.DraR1*. All primers are listed in Supplementary Table S2.

### Quantification of Modified Bases in gDNA by UHPLC-QQQ-MS/MS

The gDNA (2–4 μg) in 26 μL of nuclease-free H_2_O was denatured at 100°C for 5 min, chilled on ice for 2 min, and digested by 1 μL nuclease P1 (1U/μL, Wako United States) in 10 mM NH_4_OAc (adding 3 μL 100 mM NH_4_OAc, pH 5.3) at 42°C overnight. It was followed by addition of 3.4 μL NH_4_HCO_3_ (1 M) and 1 μL of phosphodiesterase I from *Crotalus adamanteus* venom (0.001 U, Sigma-Aldrich) at 37°C for 2 h and finally by addition of 1 U of alkaline phosphatase from *E. coli* (Takara) at 37°C for 2 h. Digested DNA was diluted twofold with nuclease-free H_2_O. The diluted solution was filtered through a 0.22 μm filter and 5 μL was injected for liquid chromatography with tandem mass spectrometry (Greer et al., 2015; Liu et al., 2016).

2′-Deoxyguanosine (dG, D7145), Thymidine (dT, T9250), 2′-Deoxyadenosine (dA, D7400), 2′-Deoxycytidine (dC, D3897), and *N*^6^-Methyl-2′-deoxyadenosine (m^6^dA or 6mA, M2389) were purchased from Sigma. 2′-Deoxy-5-methylcytidine (m^5^dC or 5mC, D3610) was purchased from Tokyo Chemical Industry (TCI, Japan). 2′-Deoxy-*N*^4^-methylcytidine (m^4^dC or 4mC) was a generous gift from Prof. Jianzhao Liu (Zhejiang University, China). Nucleosides were separated by reversed-phase UHPLC on a C18 column with online mass spectrometry detection using a Waters TQ MS triple-quadrupole LC mass spectrometer in positive electrospray ionization mode (Waters, Milford, MA, United States). The elution program was shown as following: the C18 column was washed using ddH_2_O and equilibrated with 98% solvent A (1‰ Formic acid/ddH_2_O) and 2% solvent B (1‰ Formic acid/methanol); the content of B was increased gradually from 2 to 100% in 5 min after injection, and subsequently continue to elute 2.5 min using 100% B; and then the column was treated circularly as the first two steps did before the next injection. Nucleosides were quantified using nucleoside precursor ion to base ion mass transitions of 228.2–112.1 for dC, 242.2–126.1 to separate m^4^dC and m^5^dC by retention time, 266.1–150.0 for m^6^dA and 252.1/136.0 for dA (Yu et al., 2015; Liu et al., 2016). Ratio quantification was performed using calibration curves obtained from nucleoside standards run at the same time.

### Methyltransferase Cloning

The *M.DraR1* gene was amplified by PCR using the primers M.DraR1-pRRS-F and M.DraR1-pRRS-R and cloned into the pRRS plasmid as described previously (Clark et al., 2012). The pRRS-M.DraR1 plasmid was transformed into *E. coli* DH5α cells and cultured on ampicillin-resistant LB plates for 20 h at 37°C. Clones were selected and confirmed by DNA sequencing using the sequencing primers pRRS-F and pRRS-R.

The constructed pRRS-M.DraR1 vector was transferred to *E. coli* ER2796 cells. Plasmid DNA was isolated and the methylation status was assessed by digestion with *Hin*dIII and a relevant cognate REase *Sac*II. The unmethylated control substrate was produced by PCR amplification using primers pRRS-F and pRRS-F-R. Null pRRS was analyzed as well. Additional REases, including *Pvu*II, *Hha*I, *Hpa*II, and *Hae*III were also used to digest the substrates. Samples were separated by 5% native polyacrylamide gel electrophoresis (PAGE). Gels were stained using ethidium bromide (EB) and bands were visualized using an Automatic Digital Gel Image Analysis System (Tannon 3500, Shanghai, China). The pRRS plasmid and *E. coli* ER2796 host strain were gifts from Sir Richard J. Roberts (New England Biolabs, Ipswich, MA, United States). All primer sequences are listed in Supplementary Table S2. All restriction enzymes were purchased from New England Biolabs, United States.

### Expression and Purification of M.DraR1 MTase

The *M.DraR1* gene was amplified and cloned into a modified pET28a expression vector (Zhao et al., 2015). After confirmed by DNA sequencing, the constructed plasmid was transformed into ER2566 strain. Protein expression was induced at 16°C by adding isopropyl-β-D-thiogalactopyranoside (IPTG) to a final concentration of 0.4 mM.

The M.DraR1 protein was purified using GE Akta Pure 25 system (GE Healthcare, United States). Briefly, cells resuspended in lysis buffer (20 mM Tris–HCl, pH 8.0; 500 mM NaCl; 5% (w/v) glycerol; 3 mM β-mercaptoethanol and 9 mM imidazole) were lysed by sonication and clarified by centrifugation at 20,000 g for 30 min at 4°C. The supernatant was purified using a HisTrap HP column (GE Healthcare) equilibrated with buffer A (20 mM Tris–HCl pH 8.0; 500 mM NaCl; 5% (w/v) glycerol and 3 mM β-mercaptoethanol), washed with 40 mM imidazole and eluted with 250 mM imidazole. After TEV tag removal using TEV protease, the protein was loaded onto an MBPTrap HP column (GE Healthcare) to remove His-MBP and uncleaved proteins. The flow-through fractions were collected, desalted using a HiPrep^TM^ 26/10 column (GE Healthcare) with buffer B (20 mM Tris–HCl, pH 8.0; 100 mM NaCl; 1 mM dithiothreitol; 5% (w/v) glycerol and 1mM EDTA) and loaded onto a Heparin HP column (GE Healthcare) pre-equilibrated with the same buffer. Fractions containing M.DraR1 protein were eluted using a linear NaCl gradient from 100 to 1000 mM. Finally, the protein was purified using a Superdex 200 10/300 GL column (GE Healthcare) with buffer C (20 mM Tris–HCl, pH 8.0 and 200 mM KCl). The purified protein was assessed by sodium dodecyl sulfate-PAGE, Western blotting and matrix-assisted laser desorption/ionization time of flight mass spectrometry.

### Electrophoresis Mobility Shift Assays

Electromobility shift assays were performed as previously described with some modifications (Cheng et al., 2016). Briefly, 20 μL reaction mixtures containing 100 μM double-stranded DNA (generated by annealing the oligonucleotides listed in Supplementary Table S2) and various concentrations of purified M.DraR1 (0, 100, 200, 300, 400, 500, 600, and 1000 μM) in binding buffer (20 mM Tris–HCl, pH 8.0 and 100 mM KCl) with excess or no SAM, were incubated for 60 min at 4°C. Samples were analyzed by 12% native PAGE gel electrophoresis and bands were visualized by EB staining. All DNA oligonucleotides were purchased from Sangon.

### *In vitro* M.DraR1 Protection Assays

For *in vitro* methylation, 1 μg unmethylated pRRS-M.DraR1 plasmid, 2 μg DNA containing three “CCGCGG” sites (PCR products of λDNA and gDNA of *D. radiodurans*, 2,836 and 1,318 bp, respectively, primers listed in Supplementary Table S2) and λDNA were incubated with 1 μM purified M.DraR1 at 30°C for 1 h in reaction buffer (20 mM Tris–HCl, pH 8.0; 100 mM KCl; 0.1 mM EDTA; 3 mM β-mercaptoethanol and 80 μM SAM). Methylated products were then purified and digested as described above. Finally, the digested products were analyzed by 1% agarose or 5% native PAGE gel electrophoresis as described above.

### Growth Curve Test

Growth curve and phenotypic assays were performed as previously described (Cheng et al., 2015). *D. radiodurans* and derivative strains were grown to early exponential phase (OD_600_ ≈ 1.0) and chilled on ice. To assess growth rate, 0.5 mL aliquots were resuspended in 50 mL fresh TGY media and incubated at 30°C with shaking at 220 rpm. The OD_600_ was measured every 4 h for a total incubation period of 40 h. Growth curves were created from at least three independent measurements using GraphPad Prism (GraphPad Software, Inc., La Jolla, CA, United States).

### Transformation, Recombination, and Spontaneous Mutation Frequency

A total of 0.2 μg of each of the shuttle vector pRADK, non-methylated and methylated pRADKm (containing one “CCGCGG” site inserted by site-directed mutagenesis using primers pRADKm-F and pRADKm-R and confirmed by sequencing using primer pRADKm-seq) was transferred into *D. radiodurans* strains. After incubation, cultures were plated onto TGY and TGY containing chloramphenicol (TC) plates. The transformation frequency was determined by dividing the number of transformant colonies growing on TC plates by the total number of viable cells on TGY plates (Cheng et al., 2015). We also amplified a donor DNA fragment containing a kanamycin resistance gene from Δ*crtB* mutant (Han et al., 2017). A total of 0.5 μg this fragment was used to measure the recombination capacity of the Δ*M.DraR1* mutant. Approximately 10^9^ freshly cultured cells were harvested at the early exponential phase, resuspended and plated on TGY plates containing rifampicin to determine the number of mutants and on native TGY plates to determine the total number of viable cells (Cheng et al., 2015). Three replicates were performed for each strain.

### RNA Isolation and Sequencing (RNA-Seq)

Total RNA of two biological replicates (OD_600_ = 0.5) from each group was isolated using the TRIzol method according to the manufacturer’s protocol (Ambion, Foster City, CA, United States). RNA degradation and contamination were assessed using 1% agarose gels. RNA concentration and RNA integrity were assessed using an RNA Nano 6000 Assay kit and Bioanalyzer 2100 system (Agilent Technologies, Santa Clara, CA, United States). A total of 3 μg RNA per sample was used as the input material for library preparation. Index codes were added to attribute sequences to each sample. The library preparations were sequenced on an Illumina Hiseq platform and paired-end reads were generated. The raw reads were deposited in the Sequence Read Archive (SRA) database of NCBI (PRJNA532990).

### RNA-Seq Data Analysis

The index of the reference genome (downloaded directly from the NCBI) was built and clean reads were aligned to the reference genome by Bowtie2-2.2.3 (Langmead and Salzberg, 2012). HTSeq v0.6.1 was used to count the number of reads mapped to each gene. Differential expression analysis was performed using the DEGs analytical method (1.18.0), which provides statistical routines for determining differential expression in digital gene expression data using a negative binomial distribution model (Anders and Huber, 2010). GO enrichment analysis of DEGs was performed using the GOseq R package, which corrected for gene length bias. GO terms with corrected *p*-values < 0.05 were considered significantly enriched (Young et al., 2010). We also test the enrichment of DEGs in Kyoto Encyclopedia of Genes and Genomes pathways (Mao et al., 2005).

### Experimental Validation

The reliability of the RNA-seq data was verified using 13 DEGs with different fold-change values (seven up-regulated and six down-regulated genes). Total RNA was extracted and cDNA was amplified using SYBR Premix Ex Taq^TM^ (TaKaRa). RT-qPCR assays were performed using the Mx3005^TM^P Real-time detection system (Agilent, United States) and *DR_1343* was used to normalize expression. RT-qPCR primers are listed in Supplementary Table S2.

## Data Availability

The RNA−seq data from this study has been submitted to the NCBI Gene Expression Omnibus with an accession number of GSE134934 (https://www.ncbi.nlm.nih.gov/geo/query/acc. cgi?acc=GSE134934).

## Author Contributions

YH conceived the project. SL, YZ, and BT designed the experiments and wrote the manuscript. SL, HL, and JC carried out the construction, purification, mass spectrometric analysis, and biochemical experiments. SD, JH, and SM carried out the mutants and phenotypic assays. SL, LW, and XH carried out the SMRT-seq, RNA-seq, and data analysis. HX and SL performed the enzymatic activity assays. All authors took part in the data analysis and approved the final version of the manuscript to be published.

## Conflict of Interest Statement

The authors declare that the research was conducted in the absence of any commercial or financial relationships that could be construed as a potential conflict of interest.

## Footnotes

1 http://www.pacb.com/devnet/code.html
