# Supplementary material for: N4-Cytosine DNA Methylation Is Involved in the Maintenance of Genomic Stability in Deinococcus radiodurans
Source: Front Microbiol. 2019 Aug 21;10:1905. doi: 10.3389/fmicb.2019.01905 (PMC6712171; doi:10.3389/fmicb.2019.01905)
Supplement: Supplementary file 1 [file Data_Sheet_1.pdf]

Supporting information

N^4-cytosine DNA methylation is involved in the maintenance of genomic stability in *Deinococcus radiodurans*

Shengjie Li[1], Jianling Cai[1], Huizhi Lu[1], Shuyu Mao[1], Shang Dai[1], Jing Hu[1], Liangyan Wang[1], Xiaoting Hua[2], Hong Xu[1], Bing Tian[1], Ye Zhao[1] and Yuejin Hua[1*]

[1] The MOE Key Laboratory of Biosystems Homeostasis & Protection, Zhejiang University, Hangzhou, China

[2] Department of Infectious Diseases, Sir Run Run Shaw Hospital, College of Medicine, Zhejiang University, Hangzhou, China

*Correspondence:

Prof. Yuejin Hua

yjhua@zju.edu.cn

Supplementary Table 1. Strains and plasmids used in this experiment.

Supplementary Table 2. Primers or oligonucleotides used in this study.

Supplementary Table 3. The loci, protein accession numbers and recognition sequences of the annotated R-M enzymes in *D. radiodurans* R1.

Supplementary Table 4. Analysis information of MALDI-TOF/TOF MS.

Supplementary Figure 1. Restriction-modification systems in *D. radiodurans* R1.

Supplementary Figure 2. Example MS Spectra, Related to Figure 2.

Supplementary Figure 3. Multiple sequence alignments of M.DraR1.

Supplementary Figure 4. Deletion of *M.DraR1* gene in *D. radiodurans* R1 strain.

Supplementary Figure 5. PCR analysis to confirm the other three MTases mutants.

Supplementary Figure 6. Purification and identification of M.DraR1 enzyme.

Supplementary Figure 7. M.DraR1 could not methylate CpG sites randomly *in vitro*.

Supplementary Figure 8. The biological relationship of the downregulated DEGs.

Supplementary Table 1. Strains and plasmids used in this experiment.

Strain and plasmid	Relevant feature	Reference or source
**Strains**		
D.radiodurans		
DraR1 wt	*D. radiodurans* R1 wild-type strain	ATCC13939
Δ*M.DraR1*	R1 but *M.DraR1::Str*	This study
Δ*M.DraR1*/pk-*M.DraR1*	Δ*M.DraR1* compensated with pRAD-*M.DraR1*	This study
Δ*DraR1ORF2330P*	R1 but *ORF2230P::Str*	This study
Δ*DraR1ORF14075P*	R1 but *ORF14075P::kana*	This study
Δ*DraR1ORF15360P*	R1 but *ORF15360P::kana*	This study
E. coli		
ER2796	fhuA2 Δ (lacZ)r1 glnV44 trp-31 dcm-6 his-1 zed-501::Tn10 argG6 rpsL104 dam-16::Kan xyl-7 mtl-2 metR1 mcr-62 Δ (mcrB-hsd-mrr)114	Prof. Richard J. Roberts, NEB.
ER2566	F- λ- fhuA2 [lon] ompT lacZ::T7 gene 1 gal sulA11 Δ(mcrC-mrr)114::IS10 R(mcr-73::miniTn10-TetS)2 R(zgb-210::Tn10)(TetS) endA1 [dcm]	ZonHon Biopharma, Jiangshu, China.
DH(5α)	supE44, ΔlacU169 (φ80lacZΔM15), hsdR17, recA1, endA1, gyrA96, thi-1, relA1	TransGen Biotech, Beijing, China.
**Plasmids**		
pRRS	Genbank accession number: JN569339	Prof. Richard J. Roberts, NEB.
pRRS-*M.DraR1*	pRRS ligated with full length *M.DraR1* gene	This study
pRADK	*E. coli–D. radiodurans* shuttle vector	Laboratory stock
pRADKm	Modified pRADK vector contains one 'CCGCGG' site	This study
M. pRADKm	Methylated pRADKm vector with M.DraR1 enzyme	This study
pRAD-M.DraR1	pRADK but *kana*^{r} was replaced and ligated with *M.DraR1*	This study
pET28a-HMT	pET28 plasmid modified with a Maltose Binding Protein and a TEV protease site	Austin, B.P., *et al.*
HMT-*M.DraR1*	pET28-HMT but ligated with *M.DraR1*	This study

Supplementary Table 2. Primers or oligonucleotides used in this study.

**Primers for PCR amplification and sequencing**	**Sequence (5'→3')**
M.DraR1-P1	CACCCCCGTCCAGACTCAGC
M.DraR1-P2	CGCGGATCCTGAGCTGGACTCCCGAAGTGC
M.DraR1-P3	CCCAAGCTTCCACAATCACGGGCCTAACTACAG
M.DraR1-P4	GTGCCCATCTGGAGTCGCTACC
M.DraR1-P5	GTGAACTGGATTGCGGGATT
M.DraR1-P6	TTCCGCAGGTAGTGATAGTTGTTC
M.DraR1-F	TTAATTTCATATGACGCAACCTCTTCTCTTTGACC
M.DraR1-R	TATGGATCCTTACCTGGTCAGTTCAACCACGG
pRAD-F	AATTCGGCTTGGAAGCACGTA
pRAD-R	TTGGCGTTACAAGGATGATCCA
2230P-P1	GTCGGGTTGTTCGGGTAAT
2230P-P2	TTGGATCCGAACCTCTTCAGAGTACGGCTTA
2230P-P3	ATTAAGCTTGACCCGACACTGGCGC
2230P-P4	CGAAATTCTGCGGGTGG
2230P-P5	AAGAACTGCCTGAGCGGTACA
2230P-P6	CAATGGACAGAACTTTGGATGACT
14075P-P1	ATCAACGGCGGACAAAACGG
14075P-P2	CGCGGATCCATCAGCTCGCAGGCTAGCGC
14075P-P3	CCCAAGCTTAGCTTGAGATTCGTACTCGCCTTAC
14075P-P4	TGGCGGTTAGGCTTCCTTCTG
14075P-P5	GGCACAAAGGACAAAGGGTT
14075P-P6	TCGGTCCTTGAATGTCTCCCT
15360P-P1	AGGACAACCGCATTGACACCC
15360P-P2	TTGGATCCTCAAGACAGGGCTCCAAGTTTGG
15360P-P3	ATTAAGCTTTTGACTCGTGACCAGCTGGAAGA
15360P-P4	AAGGCGGGAAGGGTTGAAGA
15360P-P5	ACGAGATTCCCGAAAAGACCG
15360P-P6	CGTCGGGAAACTCGATGTGC
M.DraR1-pRRS-F	TTTcctgcaggTTAAGGTTAATCATATGACGCAACCTCTTCTCTTTGA
M.DraR1-pRRS-R	TTTggatccCCGCGGTTACCTGGTCAGTTCAACCACG
pRRS-F	ACCCCAGGCTTTACACTTTATGCT
pRRS-R	GCACAGATGCGTAAGGAGAAAAT
pRRS-F-R	AGCATAAAGTGTAAAGCCTGGGGT
λDNA-F	TTGTGGGGTGAATATGGCAGTA
λDNA-R	CAGGCTTCCAGCAACGAGG
gDNA-F	TTTGCAGGAGCCGAAATG
gDNA-R	ACTGGCCGATGTGGTCTTGG
pRADKm-F	CCATTCTTGCAGCCGCGGTCAGGGTCTTGACGT
pRADKm-R	ACGTCAAGACCCTGACCGCGGCTGCAAGAATGG
pRADKm-seq	AATGGCTGGCCTGTTGAACAAGTCT
**Oligonucleotides for EMSA**	

S1-F	CAGGCCGCGGCT
S8-F	AGGCCGCGGCTA
S1/8-R	TAGCCGCGGCCT
S2-F	CAGGTCGCGGCT
S2-R	TAGCCGCGACCT
S3-F	CAGGCTGCGGCT
S3-R	TAGCCGCAGCCT
S4-F	CAGGCCTCGGCT
S4-R	TAGCCGAGGCCT
S5-F	CAGGCCGTGGCT
S5-R	TAGCCACGGCCT
S6-F	CAGGCCGCTGCT
S6-R	TAGCAGCGGCCT
S7-F	CAGGCCGCGTCT
S7-R	TAGACGCGGCCT
**Primers for RT-qPCR**	
C12-F	CGGTCTCGCCAACAAGGAAA
C12-R	TCTTTGGTCGCAGCCGTCA
1262-F	CCCAAAGTGGACTCCCCCG
1262-R	GGTTAGGCCGTTGGTCTGCA
2340-F	CGCCAACACCGTCAAGATCAA
2340-R	TCGTCGCCGTAGGAGTAGAAGC
0099-F	GTGAACGCAATCTGCCCTGGTA
0099-R	GTTCCATGCGGAGGGCTTTG
0423-F	GGCATCGGGCGTTACCTCTA
0423-R	CGCAACTGCTCCATCGCC
B0100-F	CGGATTTATCGGCAAGGAGGTC
B0100-R	CGTTCTCGCCGCAAAATCG
1877-F	CCGCGAGTTCGAGTTAAAGGTG
1877-R	GGTAGCGCTTGACCTGCACC
1343-F	GGCTGGTTTTCCGCATCCTC
1343-R	GTTGACCGTCAGGCTGCTTTC
0689-F	CCGTCAACGCCAAAGAGGAG
0689-R	CGGGTGCCGAAGAAATACTGTT
0690-F	ACGGCGTGTCGCAGCATAA
0690-R	GGGGCCAATGACTTCGCG
2244-F	CGAGCCTATTCCCGACACTGA
2244-R	ACGAGAAGGCGTTGCGCTC
A0188-F	GACCCGCAACAACCTGGATAA
A0188-R	GTCTTCGTCGTCCTCGGGG
1939-F	ATCCCTACGACTCCTTTGTCAACG
1939-R	ACGACCTGCTTGCCGTTTTC
A0157-F	CCCACGCTCGCCAACATCTA
A0157-R	TGCTCTTCCACTCGCCGC

Supplementary Table 3. The loci, protein accession numbers and recognition sequences of the annotated R-M enzymes in *D. radiodurans* R1.

Name	Enzymes/ORF	Type/Annotation [a]	Locus tag	Protein ID	Recognition (cleavage) [b]	Reference locus tag
Mmel	DraR1ORF2230P	Type II restriction enzyme and methyltransferase	A2G07_02230	ANC70677.1	CAAGN**<u>A</u>**C, m6A	DR_2267
Mrr	DraR1MrrP	Type II restriction enzyme	A2G07_10975	ANC72247.1	Unkown	DR_0508
Mrr	DraR1Mrr2P	Type IV Methyl-directed restriction enzyme	A2G07_10550	ANC72172.1	Unkown	DR_0587
/	DraR1ORF14075P	Putative Type IIG restriction enzyme/N6-adenine DNA methyltransferase	A2G07_14075	ANC72977.1	Unkown, m6A	DR_A0119
			A2G07_14080	ANC72978.1		DR_A0119.1
/	DraR1ORF15360P	Type IIG restriction enzyme and methyltransferase	A2G07_15365	ANC73228.1	Unkown, m6A	DR_B0137
			A2G07_15360	ANC73227.1		DR_B0138
McrB	DraR1McrBP	Type IV Methyl-directed restriction enzyme	A2G07_15330	ANC73223.1	Unkown	DR_B0143
McrC	DraR1McrCP		A2G07_15325	ANC73222.1	Unkown	DR_B0144
M. DraR1 [c]	M.DraR1ORF16000P	Type II methyltransferase, subtype: alpha	A2G07_16000	ANC73351.1	C**<u>C</u>**GCGG, m4C	DR_C0020

[a] The annotation and types of RM enzymes are presented in REBASE, a restriction enzyme database.

[b] The methylated nucleotide in the motif is shown as bold and underlined letter.

[c] The recognition sequence of M.DraR1 is confirmed in our study

Supplementary Table 4. Analysis information of MALDI-TOF/TOF MS.

Protein Name	Species	Accession No.	Protein MW	Protein PI	Pep. Count	Protein Score	Protein Score C. I. %	Total Ion Score	Total Ion C. I. %
hypothetical protein A2G07_16000	*Deinococcus radiodurans*	ANC73351.1	48526.6	6.11	22	762	100	629	100

Peptide Information

Calc. Mass	Obsrv. Mass	± da	± ppm	Start Seq.	End Seq.	Sequence	Ion Score	C. I. %	Modification	Result Type
852.4363	852.4354	-0.0009	-1	296	301	YWQTVR				Mascot
871.4196	871.4301	0.0105	12	139	145	TPFYSEK				Mascot
891.4683	891.4635	-0.0048	-5	131	138	VPQGFTSR				Mascot
1040.6211	1040.6199	-0.0012	-1	356	365	TQAVLRPGAK				Mascot
1059.6157	1059.6178	0.0021	2	408	418	IGSSIVGTGLR				Mascot
1114.5415	1114.5535	0.012	11	287	295	YLELDNYGK				Mascot
1192.6572	1192.6638	0.0066	6	425	434	LYEAVVELTR				Mascot
1192.6572	1192.6638	0.0066	6	425	434	LYEAVVELTR	77	100		Mascot
1636.9091	1636.9043	-0.0048	-3	213	227	LLEMHADLLGVQGIK				Mascot
1642.8007	1642.8088	0.0081	5	228	242	LGGQTAQVYQGSFMR				Mascot
1642.8007	1642.8088	0.0081	5	228	242	LGGQTAQVYQGSFMR	95	100		Mascot
1658.7955	1658.807	0.0115	7	228	242	LGGQTAQVYQGSFMR			Oxidation (M)[14]	Mascot
1856.9171	1856.9246	0.0075	4	193	210	AAAGKPDIEDADVAQVMR				Mascot
1880.031	1879.9791	-0.0519	-28	211	227	DKLLEMHADLLGVQGIK				Mascot
1903.9813	1903.9896	0.0083	4	268	283	NTRPHLYWLGYATSPK				Mascot
1903.9813	1903.9896	0.0083	4	268	283	NTRPHLYWLGYATSPK	49	100		Mascot
2041.0865	2041.0918	0.0053	3	79	96	GHSVVSYDINPFPLLVQR				Mascot
2041.0865	2041.0918	0.0053	3	79	96	GHSVVSYDINPFPLLVQR	149	100		Mascot

2070.0291	2070.0288	-0.0003	0	150	166	VLHVWDFINEVADEDLR				Mascot
2432.0579	2432.0686	0.0107	4	331	351	GVYGGQGWANYATEYFNDTYR				Mascot
2880.4712	2880.4604	-0.0108	-4	1	25	MTQPLLFDLPTPRPTYRDTAFASNK				Mascot
2889.3401	2889.3694	0.0293	10	167	192	DLFQVAFGATMVSYSNYSYEPSLGSR				Mascot
2910.3979	2910.415	0.0171	6	243	267	SELPDSSVDLMVTSPPYLNNYHYLR	84	100		Mascot
2926.3928	2926.4099	0.0171	6	243	267	SELPDSSVDLMVTSPPYLNNYHYLR			Oxidation (M)[11]	Mascot
2928.4561	2928.4287	-0.0274	-9	101	128	AIQDVTPAEFAQQIEAFTAHMATGGVPK				Mascot
2948.3638	2948.416	0.0522	18	331	355	GVYGGQGWANYATEYFNDTYRFLQK				Mascot
2993.5005	2993.4846	-0.0159	-5	305	330	YQTSLIFDSPWLQDLVNQLAGVQSDR				Mascot
3141.5688	3141.5815	0.0127	4	377	404	GTNLPIDEVFTHIAQHLGFSGHDIHMVR				Mascot
3157.5637	3157.5732	0.0095	3	377	404	GTNLPIDEVFTHIAQHLGFSGHDIHMVR			Oxidation (M)[26]	Mascot

Supplementary Figure 1

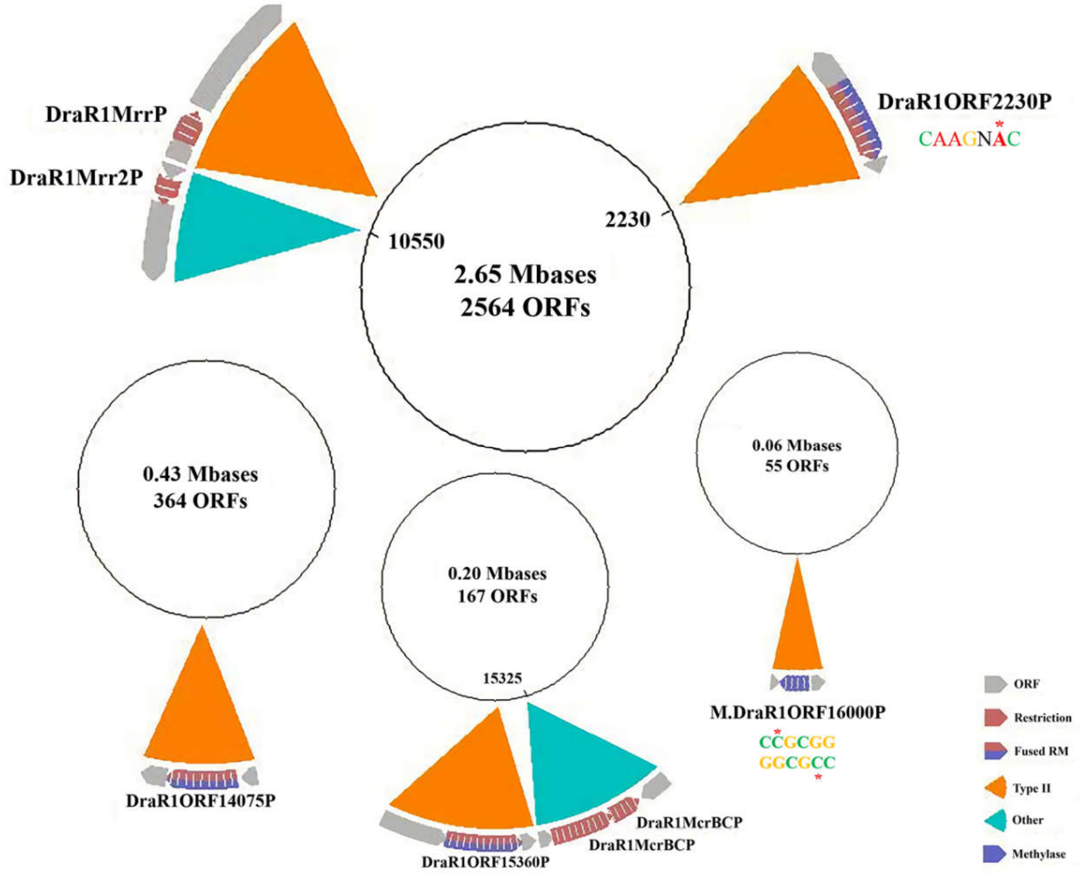

Supplementary Figure 1. Restriction-modification systems in *D. radiodurans* R1. Three fused polypeptides containing both DNA methyltransferase and endonuclease activity are located in the chromosome I, chromosome II and the large plasmid. Four restriction endonucleases, two Mrr and two McrBC types, are at the chromosome I and large plasmid, respectively. A putative methylase is presented in the small plasmid.

Supplementary Figure 2

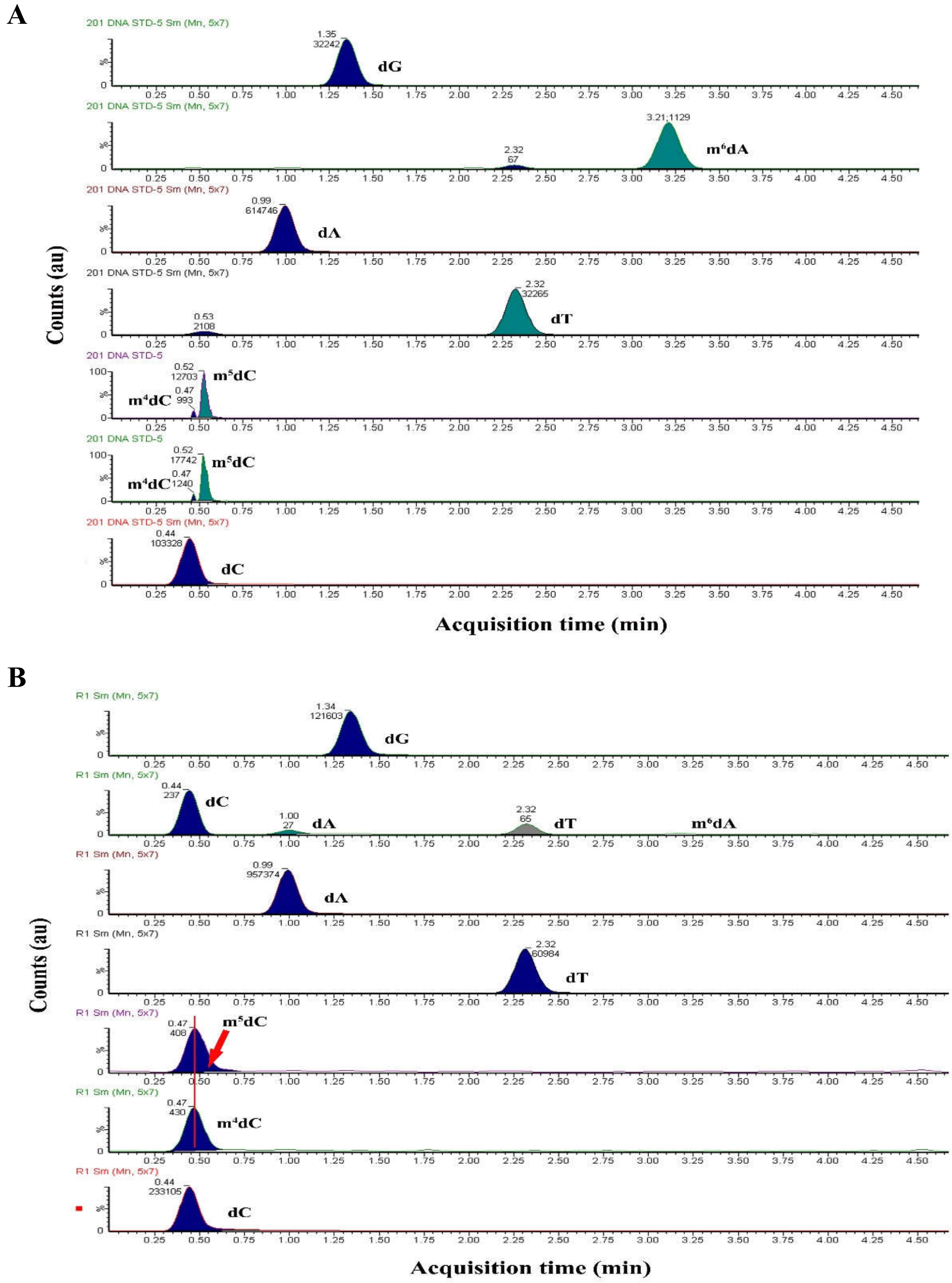

Supplementary Figure 2. Example MS Spectra, Related to Figure 2.

(A) Nucleoside standards representing all different bases including 4mC, 6mA and 5mC.

(B) Representative MS spectra of *D. radiodurans* R1 genomic DNA. These spectra demonstrate where levels of 6mA and 5mC (red arrow showed) were extremely low compared to 4mC. There are slight variations from run to run due to column and flow rate differences but the peak order is consistent.

Supplementary Figure 3

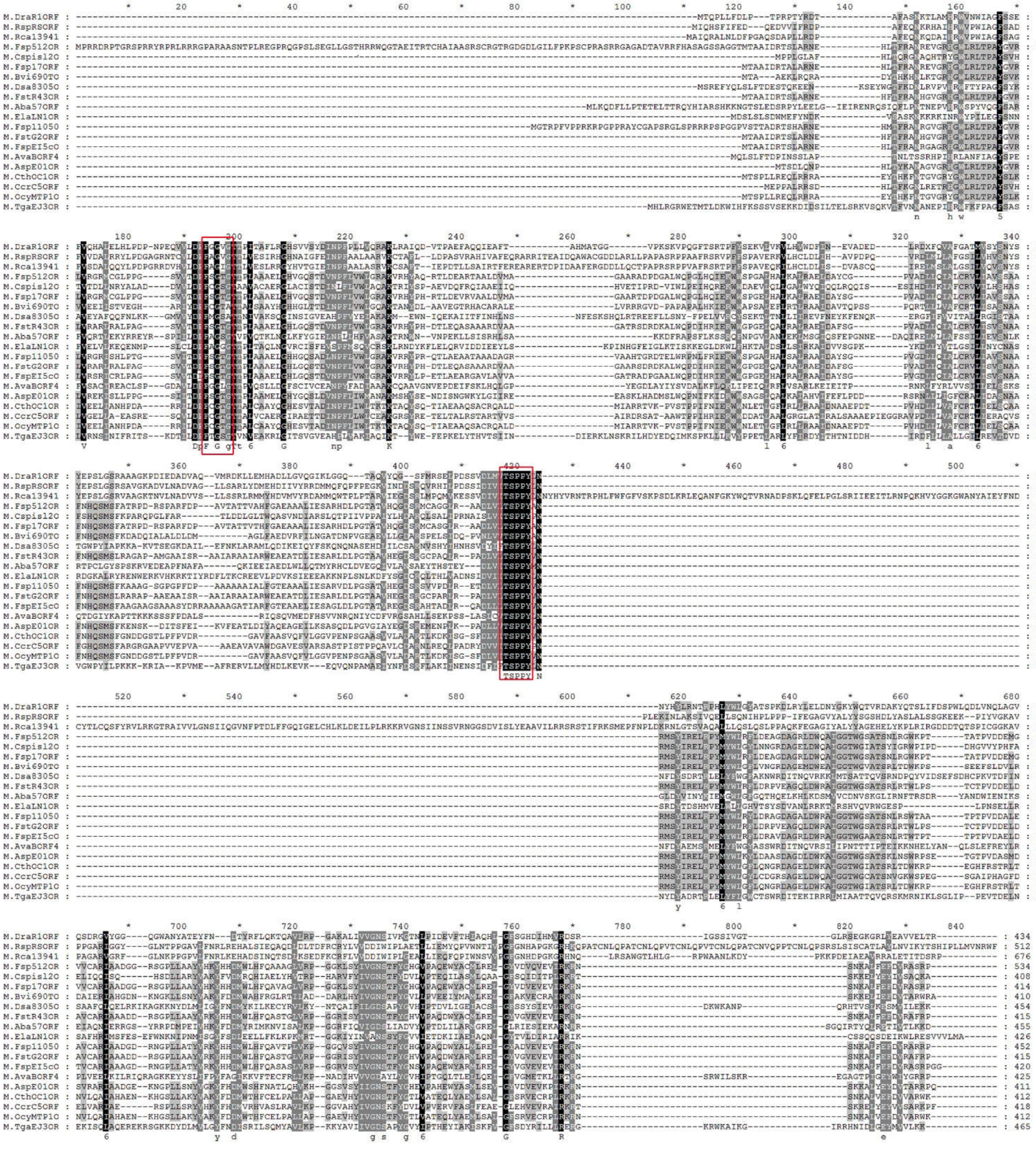

Supplementary Figure S3. Multiple sequence alignments of M.DraR1.

Sequences are from the top 20 recorded hits using BLASTP tool in REBASE. The query sequence is M.DraR116000P from *D. radiodurans* R1. The conserved N-terminal SAM-binding motif (‘FxGxG’) and C-terminal catalytic motif (‘SPPY’) are indicated by red solid line boxes. Identical residues are shown as white letters with black background, and similar residues are shown as white letters with gray background.

Supplementary Figure 4

A

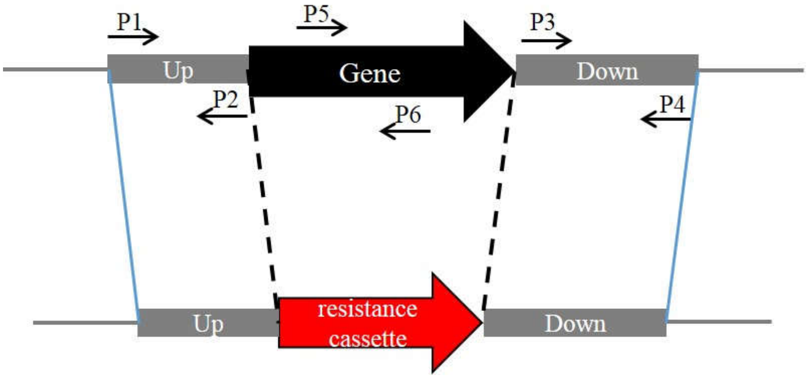

B

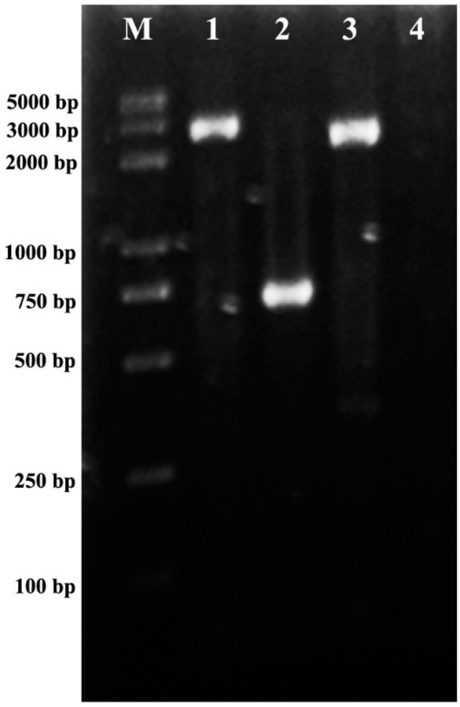

Supplementary Figure 4. Deletion of *M.DraR1* gene in *D. radiodurans* R1 strain.
(A) Scheme of gene mutation by homologous recombination which replaced the targeted ORFs with antibiotic resistant fragment. P1, P2, P3, P4, P5 and P6 refer to the primer pairs (Supporting information, supplementary table 2).
(B) PCR analysis to confirm the mutation of *M.DraR1* strain. The amplicon from the mutant (P1/P4 primers, 2648 bp, lane 3) is shorter than that of the wild type (3062 bp, lane 1), indicating that *M.DraR1* was replaced with the streptomycin-resistance fragment. Further, an interior DNA fragment of this gene was detected by amplification using primers P5/P6. No products corresponding to the size of the interior fragment from wild type (706 bp, lane 2) was observed in the mutant (lan 4), suggesting that the wild type alleles had completely replaced by streptomycin-resistance fragment in the mutant.

Supplementary Figure 5

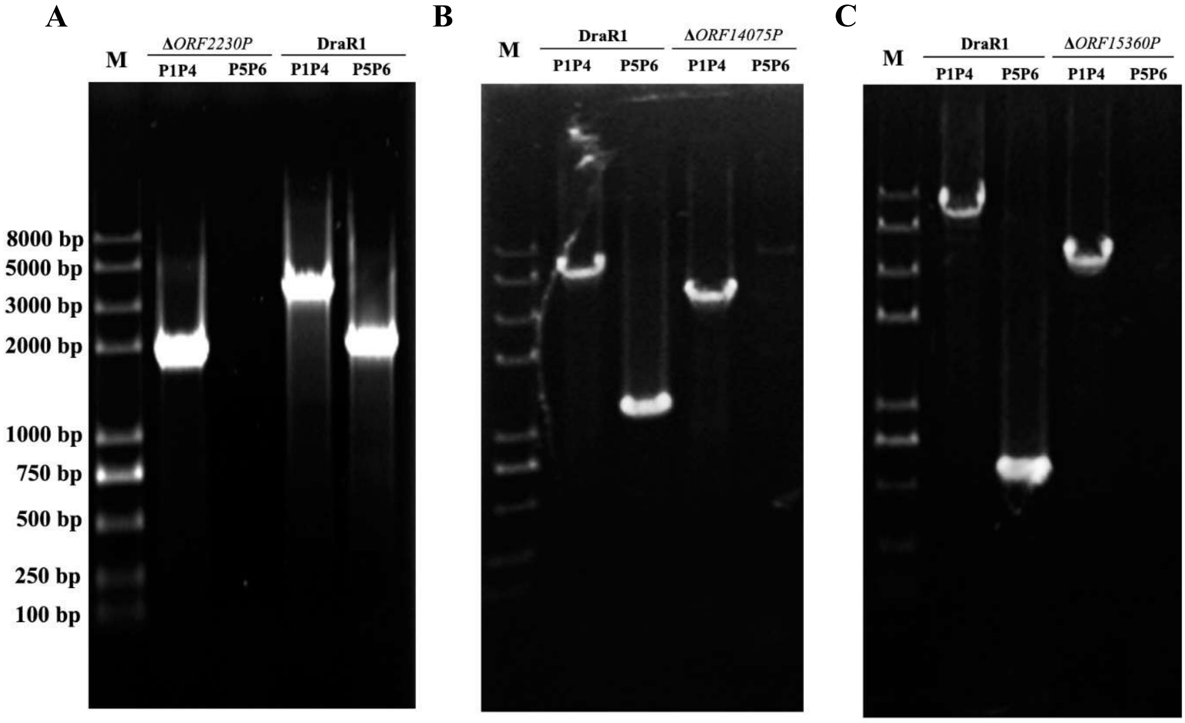

Supplementary Figure 5. PCR analysis to confirm the other three MTases mutants. (A) PCR analysis to confirm the mutation of ORF2230P. The amplicon from the mutant (P1/P4 primers, 1914 bp) is shorter than the amplicon from the wild type (P1/P4, 3555 bp). Further, no products corresponding to the size of the interior fragment from wild type (2038 bp) was observed in the mutant, suggesting that the wild type alleles had completely replaced by streptomycin-resistance fragment in the mutant.
(B) PCR analysis to confirm the mutation of ORF14075P. The corresponding amplicon from the mutant (P1/P4 primers, 3296 bp) is shorter than the amplicon from the wild type (P1/P4, 4906 bp). Further, no products corresponding to the size of the fragment from wild type (1358 bp) was observed in the mutant, suggesting that the wild type alleles had completely replaced by kanamycin resistance fragment in the mutant.
(C) PCR analysis to confirm the mutation of ORF15360P. The corresponding amplicon from the mutant is 3270 bp (P1/P4 primers), shorter than that of the wild type (5530 bp). Further, no products corresponding to the size of the interior fragment from wild type (522 bp) was observed in the mutant, suggesting that the wild type alleles had completely replaced by kanamycin resistance fragment in the mutant. Primers were listed in supplementary table 2 .

Supplementary Figure 6

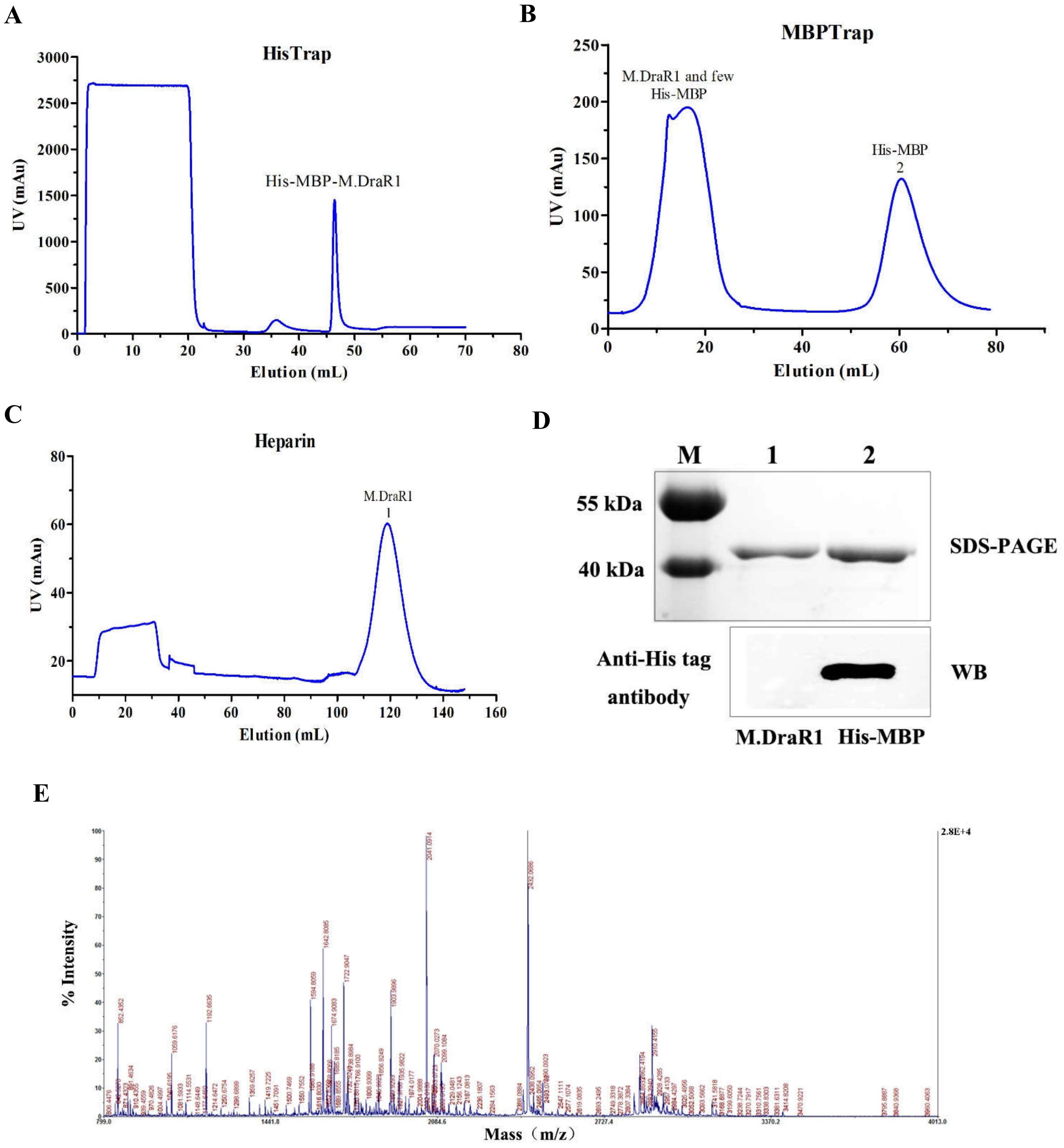

MTQPLLFDLPTPRPTYRDTAFASNKTLAMHRWVNWIAGFSSEFVQHALELHLPDPNPEQVVLDP<u>FGGVG</u>TTPITAFLR
GHSVVSYDINPFPLLVQRAKLRAIQDVTPAEFAQQIEAFTAHMATGGVPKSKVPQGFTSRTPFYSEKVLVKVLHVWDFI
NEVADEDLRDLFQVAFGATMVSYSNYSYEPSLGSRAAAGKPDIEDADVAQVMRDKLLEMHADLLGVQGIKLGGQTAQ
VYQGSFMRSELPDSSVDLMVT<u>SPPY</u>LNNYHYLRNTRPHLYWLGYATSPKDLRYLELDNYGKYWQTVRDAKYQTSLIFD
SPWLQDLVNQLAGVQSDRGVYGGQGWANYATEYFNDTYRFLQKTQAVLRPGAKALIVVGNSIVKGTNLPIDEVFTHIA
QHLGFSGHDIHMVRDSRIGSSIVGTGLRSEGKGRLYEAVVELTR

F

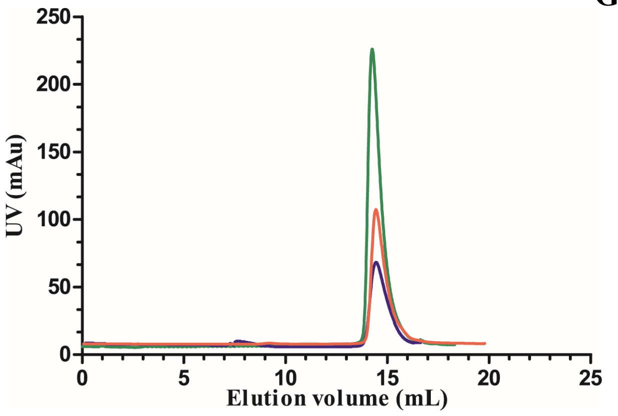

G

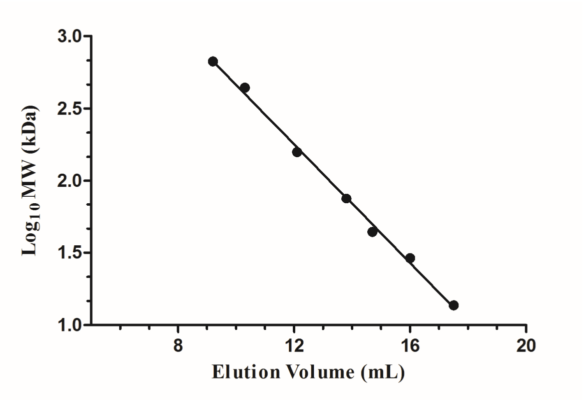

Supplementary Figure 6. Purification and identification of M.DraR1 enzyme.

(A) Representative diagrams of protein purification by HisTrap. The fused His-MBP-M.DraR1 protein was eluted with 250 mM imidazole.

(B) Representative diagrams of MBPTrap. After TEV protease cleaved, the protein was loaded onto an MBPTrap column to remove His-MBP and uncleaved proteins (Peak 2). The flow-through fractions containing M.DraR1 and few His-MBP protein were collected.

(C) Representative diagrams of HiTrap Heparin. The collected proteins from MBPTrap column were desalted and loaded in Heparin column. Fractions containing M.DraR1 protein were eluted using a linear NaCl gradient (Peak 1).

(D) Western blot analysis was used to distinguish M.DraR1 (Peak 1) from His-MBP (Peak 1) using anti-his tag antibody.

(E) Peptide mass fingerprint (PMF) of M.DraR1 protein. The identified peptides are shown in bold red text and detailed information was shown in supplementary table 4. FxGxG and SPPY conserved motifs were shown in underline.

(F) Gel-filtration analysis revealed that M.DraR1 exist as a monomer in solution. FPLC system coupled to a Superdex 200 10/300 GL column. Elution profles at 280 nm are different concentration of M.DraR1 protein. Purple, 0.1 mg/mL; Orange, 0.2 mg/mL; Green, 0.5 mg/mL.

(G) Protein size standard curve created with ribonuclease A (13.7 kDa), carbonic anhydrase (29 kDa), ovalbumin (44 kDa), conalbumin (75 kDa), aldolase (158 kDa), ferritin (440 kDa), and thyroglobulin (669 kDa).

All peaks were reconstructed using GraphPad Prism software.

Supplementary Figure 7

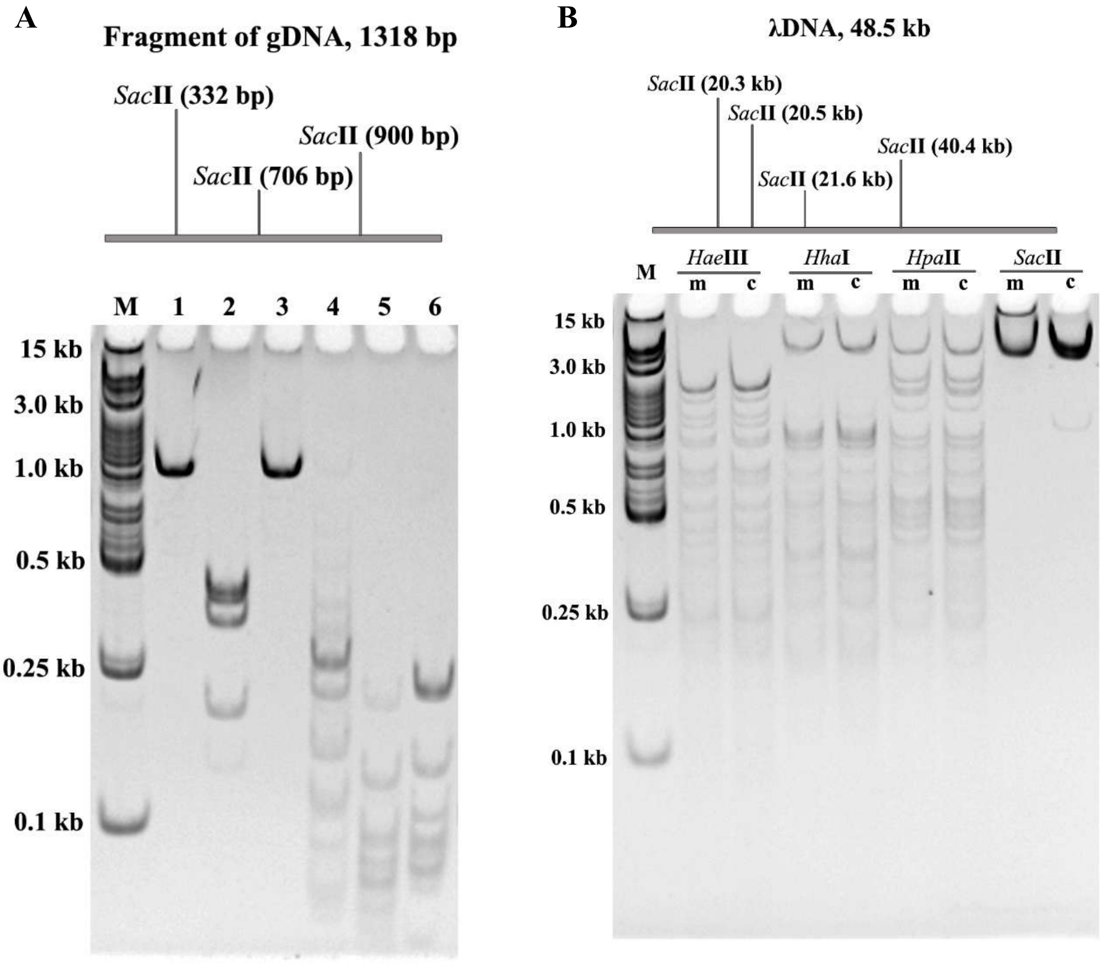

Supplementary Figure 7. M.DraR1 could not methylate CpG sites randomly *in virto*.

(A) The methylation of DNA fragment with M.DraR1 could not block the activities of *Hae*III (lane 4), *Hha*I (lane 5) and *Hpa*II (lane 6) contrasting to *Sac*II (lane 3). Lane 1 stands for the amplified PCR fragment containing three 'CCGCGG' sites form gDNA of DraR1. The unmethylated control one was digested to four bands by *Sac*II (lane 2).
(B) The unmethylated (c) and methylated (m) λDNA showed the same digestive profiles by *Hae*III, *Hha*I and *Hpa*II. M, 250 bp DNA ladder (TSJ105-100) from Beijing TsingKe Biotech Co., Ltd. All experiments were performed in three independent biological replicates.

Supplementary Figure 8

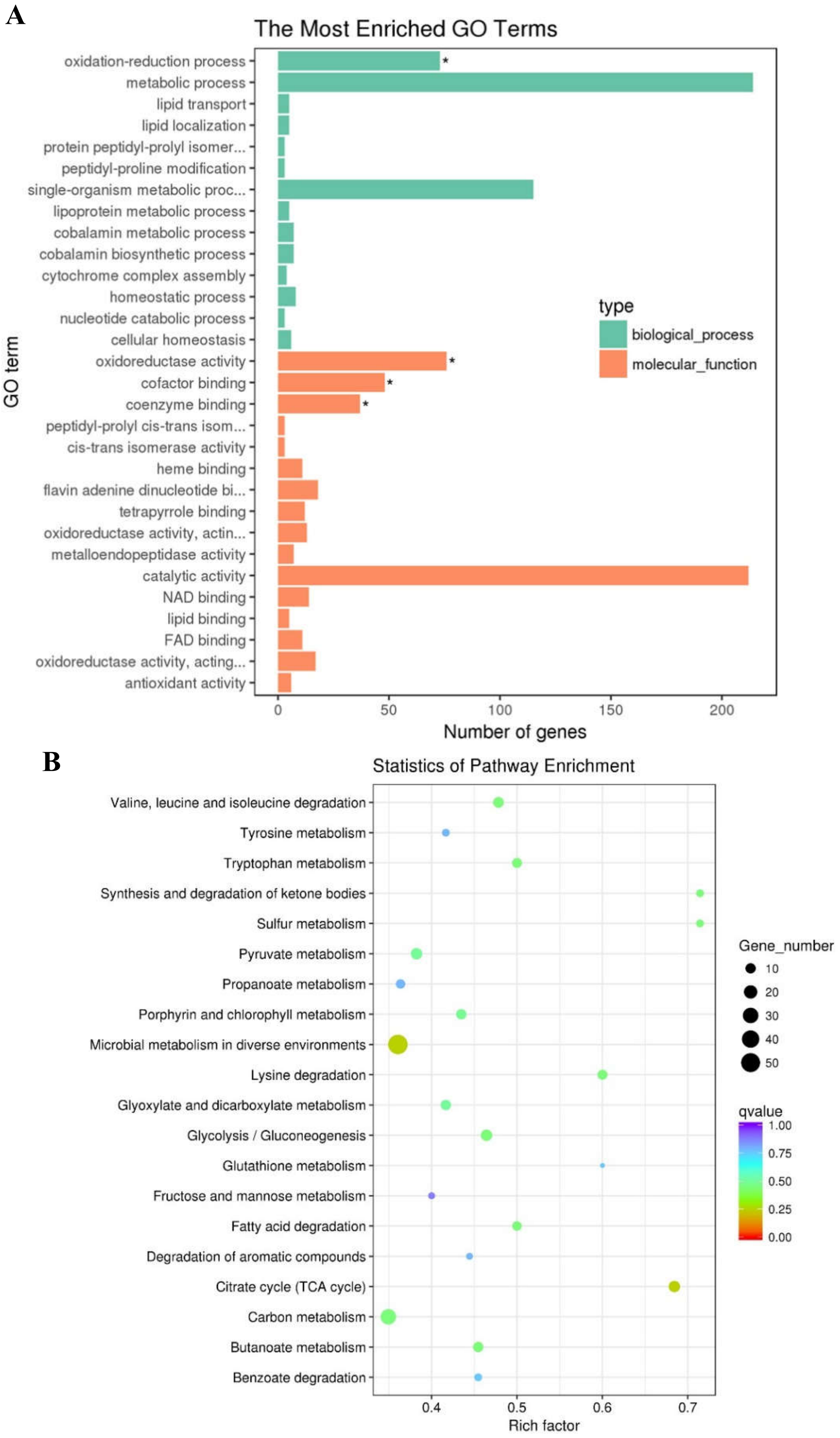

Supplementary Figure 8. The biological relationship of the downregulated DEGs.
(A) Functional categories of the downregulated DEGs in the GO database. Bars with asterisks represent significantly enriched terms ($p < 0.05$).
(B) Functional categories of the downregulated DEGs in KEGG pathways.
